# BABINE: An original and user-friendly scale for the simple and quick management of herb-drug interactions in clinical practice

**DOI:** 10.1186/s12906-024-04706-9

**Published:** 2024-12-18

**Authors:** Anthony Cnudde, Camille Allely, Natacha Biset, Pierre Champy, Nathalie Fouilhé, Fanny Huret, Sibi Lawson, Aline Mercan, Doris Pascale Noukela Noumi, Serge Michalet, Andrea Montis, Stephanie Pochet, Audrey Schils, Cecilia Tangeten, Michel Tod, Pierre Van Antwerpen, Audrey Vervacke, Florence Souard

**Affiliations:** 1 Department of Pharmacotherapy and Pharmaceutics, Faculty of Pharmacy, Université libre de Bruxelles (ULB), Brussels, Belgium; 2 Machine Learning Group, Faculty of Sciences, Université libre de Bruxelles (ULB), Brussels, Belgium; 3 CNHIM, Theriaque, France; 4 Équipe Chimie des Substances Naturelles, BioCIS, CNRS, Université Paris-Saclay, Orsay, France; 5 Centre Hospitalier Universitaire Grenoble Alpes, Grenoble, France; 6 Nutrition Risk Assessment Unit, French Agency for Food, Environmental and Occupational, Health & Safety (ANSES), Maisons-Alfort, France; 7 Université Libre de Bruxelles, Brussels, Belgium; 8 Hospices Civils de Lyon, and LBBE, Hôpital de la Croix-Rousse, Université Claude Bernard Lyon 1, Lyon, France; 9 RD3 Unit of Pharmacognosy, Bioanalysis and Drug Discovery, Faculty of Pharmacy, Université Libre de Bruxelles, Campus Plaine, Brussels, 1050 Belgium; 10 Pharmacognosy, Department of Drug Sciences, Institut des Sciences Pharmaceutiques et Biologiques (ISPB), UMR 5557 Ecologie Microbienne, Université Claude Bernard Lyon 1, Lyon, France; 11 Hôpital universitaire de Bruxelles - site Erasme (HUB) BE, Brussels, Belgium

**Keywords:** Her-drug interactions, Risk, Phytovigilance, Pharmacovigilance, Delphi, Decision support, Babine, Phytotherapy, Adverse event, Herbal supplemen

## Abstract

**Background:**

While more and more people tend to use herbal products thinking they are safer than conventional western medicine, the reality is other. If natural products are bio-active and possess potential therapeutic activities, then the benefit/risk balance should be considered like any other health product. Some herbs are known to have the potential to interact with patient’s treatment and to cause adverse drug reactions. While these are scarce, they are potentially harmful, and can lead to major sequels and even death in some cases. Despite these known facts, little guidelines about how to evaluate the risk of interaction and to handle them exist in literature. Notably, few scales allowing to assess the risk of a specific combination of herbs and drugs exist.

**Method:**

We propose a new scoring method BABINE (Boosting Analysis of Bibliography for herb- drug INteraction Evaluation) and discuss a scale to evaluate this risk based on iterative rounds of experts’ discussion.

**Results:**

After 6 rounds of case reports/clinical studies evaluation, we analyzed and synthesized criteria identified as important by the experts and developed a corresponding evaluation scale.

**Conclusion:**

Even if our scale greatly simplifies pharmacological events, we believe it provides a robust and transparent way to rapidly assess the risk of adverse event.

**Supplementary Information:**

The online version contains supplementary material available at 10.1186/s12906-024-04706-9.

## Introduction

### Herb-drug interactions

Herb-drug interactions (HDI) are events occurring when a drug and a herb (medicinal or not) are taken concomitantly and interact with each other. These can lead to modification of treatment effects by potentiating adverse drug reactions (ADRs) or by reducing drug efficacy. These events can occur through pharmacokinetic (PK) or pharmacodynamic (PD) mechanisms. On one hand, PK interactions occur through modification of absorption, distribution, metabolism or excretion of the drug. Most studied manifestations of PK variations are evidenced by the increase or decrease of plasmatic concentration (area under the curve(AUC) and C_max_), clearance and half-life ($\:{t}_{1/2}$) of drug compounds and, possibly, of their metabolites. On the other hand, PD interactions occur when the pharmacodynamic activity of a bioactive natural product amplifies (“additive or potentiating: synergy”), or conversely, opposes (“antagonism”) the pharmacodynamic activity of a drug compound. While the existence of HDI is well known by health practitioners, their evaluation and risk quantification still constitutes a major challenge. There are multiple reasons explaining this difficulty. One of them is the lack of knowledge of health professionals about herbal medicines; lots of them have negative perception of complementary medicines and tend to advise their patients against them to restrain from any use – possibly inducing “hidden” and undeclared self-medication. Another reason is the lack of clinical studies on herbal products and the limitations of the existing ones. Clinical studies focusing on herbs tend to include a limited number of participants only, mainly for economic reasons, thus reducing the statistical power and leading to uncertain conclusions. Last but not least, the complexity of herbal products which often include more than one herb in their composition. Yet considering that one herb alone could potentially contain hundreds of compounds, there are concerns about the variability and the quality of these products [1].

### Existing scales for ADRs/HDI evaluations

From our point of view, a major reason for the complexity of evaluation of HDI is the lack of interpretability of existing studies and case reports, as well as their applicability and generalizability to patients’ actual clinical situations. In the literature, most studied parameters are related to metabolization. While being interesting in terms of pharmacology, it is really complicated to infer these data to clinical situations. To handle this question or to clarify the issue, ADR probability and severity scales related to HDI are needed. These scales should allow to translate a given case or study into exploitable and robust information for a quick HDI management for clinicians. They will provide a score concerning the reliability of the information provided and the importance of the related effect and allow extrapolating it to new situations. A good ADR scale should permit to determine whether the ADR is caused by the drug/herb association or not, and how severe the expected effect will be in a given situation. Such scales exist in pharmacology literature. A first example is the Naranjo ADR Probability Scale that links the probability of an ADR with a drug. The Naranjo ADR probability scale uses a set of 10 closed questions that can be answered by *Yes*, *No* or *Do not know*. Each question/answer combination is associated with a score. The sum of these scores gives the probability of the ADR to be related to the drug intake [2]. Another method defined by the French Imputability Working Group uses two levels of information: A chronological criterion, including time onset of the ADR, its outcome and rechallenge; and a semiological criterion that considers the role of the drug, predisposing factors, specific investigations on the drug and other potential causes. The imputability score is obtained from the combination of both criteria. In some cases, it can be reinforced with a bibliographical score based on the existing experimental or clinical evidence [3]. These scales are adapted to single drug intakes and do not take interactions into account. They are thus not sufficient for the HDI problematic. Some scales go one step further in this direction. The Drug Interaction Probability Scale (DIPS) is a version of the Naranjo one in which questions have been adapted to the context of interactions [4]. Yet, this scale is adapted to drug-drug interactions (DDI) and still does not consider herbal products specificities.

To our knowledge, the only scale that considers HDI was proposed by De Smet in 2007. In this scale, the characteristics of the event, the type of study and the severity of the ADR are considered for the scoring [5]. In our point of view, this scale lacks criteria concerning HDI imputability of the observed herb-drug combination, and generalization to further cases. For example, the quality of the herbal product and use descriptions were not considered. A scale focused on HDI and based on both clinical severity, imputability and generalization is needed.

## Materials and methods

### Scale requirements and limitations

As the goal was to design a tool that should be easily handled by healthcare professionals who are not necessarily experts in pharmacognosy or pharmacology, we were particularly cautious regarding its practical aspects but also on the visual layout. Given the complexity of pharmacology, it implies to make some choices and simplification in the assessment process. For the time being, we have chosen to first focus only on real-life data, namely case reports and clinical studies. Indeed, handling an interaction would in ideal cases require to consider a substantial number of parameters. It is well known that given the age of the patient, his ethnic specificity or his habits, his reaction to a drug or a combination of drugs (i.e. pharmacogenomic) can highly change the conclusions [6]. An interaction that could then have significant effects on a given individual could have no effect at all on another one.

Yet, to correctly handle an interaction a priori, we should be able to generalize, to be concise and to keep the scorer attentive. We decided to apply these considerations by proposing a table score with the following statements:

- Be sufficiently precise to assign a reliable warning score to most of HDI described in clinical literature.
- Be sufficiently general in order to apply to most kinds of HDI met.
- Be user friendly without extensive knowledge of herbs and drugs pharmacology.

It must be emphasized that this scale is focused on purely textual information available in the target articles. It does not aim to give any clue on mechanistic interpretation but only assess the imputability, possibility to generalize and gravity of the described event based on description available in the article.

### Scale design process

To define the new HDI evaluation scale, a panel of experts was gathered. To handle the problem of HDI in a clinical context, we considered necessary to consult people with in-depth knowledge of:

1. Pharmacology and PK/PD parameters.
2. Pharmacognosy.
3. Pharmaco- and phytovigilance.
4. Medicine.

Based on these requirements, experts were contacted. These experts are listed as co-authors of this article.

To define the content of the scale, a process based on Delphi method has been chosen [7]. At first, the experts were given a set of 6 articles describing interactions studied through case reports (2 articles [8, 9]]), clinical studies (2 articles [10, 11]) and in-vitro and animal studies (1 article of each category [12, 13]). They were asked to write down at least 10 criteria they judged to be important to describe the HDI and assign them a score. These criteria were then gathered and synthesized to generate a first draw of the scale. The consistency between answers of all experts was then assessed and the scale was adapted a last time taking their comments into account. A total of 5 rounds were performed to reach the final state of the form. On each round, disparities in respondents’ answers were used as a basis for identifying discordant items and to fuel discussion. To evaluate the answers of the experts, an online form was developed. This form generates JSON files containing selected answers to the questions and associated scores.

### Iterative adaptation of the items

To reach a consensus among experts, the items for which disagreement was high had to be discussed in order to identify sources of ambiguity and disambiguate them. To do this, we brought together different profiles to test the method and then to optimize it. This group included 10 scorers − 2 Master of Pharmacy students, 5 PhD students (from the Faculty of Pharmacy with different profiles) and 3 Professors. These scorers first scored the articles that we provided them and participated in focus group discussion sessions (around 2/4 h) to discuss ambiguities and optimize the method. This process will not be detailed here, but a roadmap of discussions and modifications is available as supplementary material.

### Scoring form

**Fig. 1:**
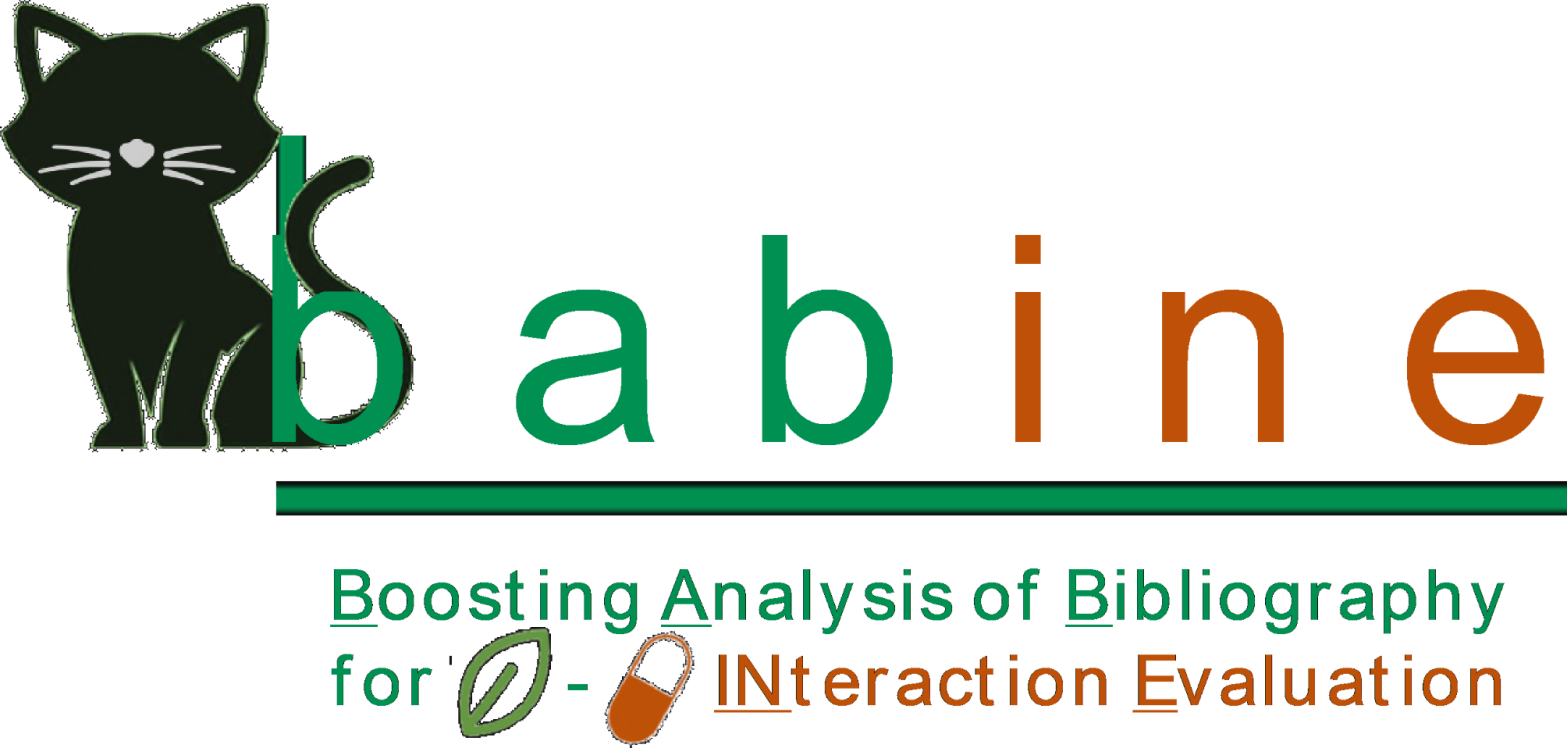
Logo with definition of the acronym babine presented on the home page of the site https://babine.ulb.be/

Once validated, the scoring form was put online from a server at our university. We also gave it a name: **BABINE** for **B**oosting **A**nalysis of **B**ibliography for herb- drug **IN**teraction **E**valuation (Fig. 1). It can be accessed at the following URL: https://babine.ulb.be.

## Results

### Keypoints in herb-drug interactions (HDI)

To evaluate literature about HDI, we first divided clinical descriptions in two general subtypes:

- Case reports, that are descriptions of interactions observed in a specific patient.
- Clinical studies, in which the interaction is investigated on a panel of participants in standardized conditions.

While other types of informative articles exist such as in vitro or animal studies, these studies require to consider many specific parameters to define a proper assessment scale. We decided to focus only on clinical data as adapting the scale to in vitro and animal data would require more than simple textual analysis of the article, which is beyond the goal of this work.

### Severity score

**Fig. 2:**
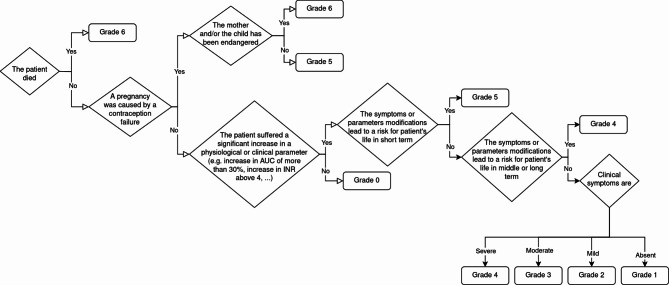
Flowchart used to assess the clinical risk when an HDI is described in a case report or clinical study. This flowchart was tested in the rounds with the scorers and validated

The first and clearer information we considered important in our scale was the severity of the observed event. To define this severity, we decided to use a separate scale based on the clinical consequences and duration of the symptoms. This gradation is based on the previous work of De Smet and on the Common Terminology Criteria for Adverse Events (CTCAE v5.0) [14]. While this later provided complete and precise description of ADRs that makes severity gradation consistent, we judged it too time-consuming to be used in our case. We still decided to use a 7 general grades system based on that of De Smet and adapt it to HDI descriptions to establish a “severity score”. The first part of the score grades gravity of the event. This score is related to clinical manifestations observed in described cases. This score is divided in 7 grades (0 to 6) and ranges from absence of symptoms (grade 0) to death (grade 6). The severity can be assessed using the flowchart described in Fig. 2.

### Imputability/generalization score

On the other hand, having noted the limitations of the literature describing HDI, we thought as appropriate to set up an “imputability/generalization score” based on key points that were more or less precisely described in the assessed publications. This imputability/generalization score reflects the level of confidence regarding the link between the observed events and the herb-drug combination given information available in the article, and how it can be generalized to further cases.

To investigate imputability and generalization in case reports and clinical studies, experts defined four key points that were judged necessary to handle an HDI:

**Information about the herb**, including the kind of preparation, specifications and / or composition.

The **proper use of the herb**: indeed, if consumption fails to follow recommendations by exceeding recommended doses or by exposing specific population (e.g. pediatric population), or if the use is recreational or specific to a geographic area, interpretation is harder to generalize.

The **mechanism of HDI**, the PK/PD/clinical parameters described and pharmacogenetics, chronological and statistical considerations.

The **patient description** (for case reports) or **cohort** (for clinical study), describing the clinical status of the subjects, and the composition of the cohort in case of clinical studies (with the possible and classic biases observed).

These four points were investigated to identify the necessary questions that needed to be answered when analyzing the interaction, in order to score it. Thanks to both severity and imputability/generalization scores described above, we established a final score to quickly inform health practitioners.

#### Herb

As mentioned above, in order to assess imputability and generalization, we first analyzed the elements that describe the herbal product used. The criteria used to evaluate herbal products description in the case reports or clinical studies are shown in Table 1 (a) and Table 2 (a). One criterion concerns the number of herbs taken simultaneously by the patient. As a result, we decided to use this information because interpreting HDI when multiple herbs are taken at the same time becomes hazardous. If 3 herbs or more are taken at the same time, we consider the HDI not interpretable to stick with our aim to make a general and user-friendly scale. This choice was made considering the literature on drug-drug interactions [15]. If the publication describes an HDI involving 3 or more herbs, the complete herb section is considered as not interpretable, and the score is 0. Consequently, the scorer does not have access to the other questions in the section.

**Table 1:** Criteria for the quotation of the imputability and generalization in case reports describing an HDI. The values for each of the lines were assigned after the first round. These values were discussed and adapted during the different runs to reach a consensus. Answers with a * automatically deactivate the subsequent questions

*N*°	Question	Points		
		Yes	No	NA
(A) Herbs				
1	The study concerns 3 herbs or more	NA*	0	/
2	The name of the supplement or herbal drug is mentioned	2	NA*	/
3	The study concerns a single molecule usually present within a mixture mix in the herb	1	NA*	/
4	The name of the herb leaves no ambiguity or the binomial Latin name is specified	1	-1	/
5	The herb part used is mentioned	1	-1	/
6	The extraction method is mentioned	1	-1	/
(B) Mechanism				
1	The study describes an interaction	0	4	/
2	The event is a pharmacodynamic event	5	0	/
3	The event is a pharmacokinetic event	3	0	/
4	The study describes the implication of a single enzyme or transporter and the drug is a substrate of this enzyme or transporter only	2	-2	0
5	The metabolization implies CYP2D6, 2C9 or 2C19	1	-1	0
6	The study concerns a single intake of the herb	3	2	0
7	A dechallenge or rechallenge is mentioned	3	0	0
(C) Patient				
1	The case concerns a patient aged 70 or more, 18 or less, or a pregnant woman	-2	0	-2
2	The treatment is directly related to the pathology of a patient with a transplant, heart/kidney/liver failure, cancer or neurological disorders or immunosuppressed	+ 2	-2	0
3	The patient’s treatment was well balanced before introduction of the herb	+ 2	0	0
(D) Proper use				
Supplement	The patient exceeded dosage recommended by the manufacturer	-2	1	-1
Essential oil	The patient took more than 10 drops a day	-2	1	-1
Juice	The patient drank more than 500mL a day	-2	1	-1
Powder	The patient took more than one teaspoon a day	-2	1	-1
Herbal tea	The patient drank more than 1 L a day	-2	1	-1
Recreational	The case concerns a recreational use	-2	1	-1
Other	The patient did not respect manufacturer recommendations	-2	1	-1

**Table 2:** Criteria for the quotation of the imputability and generalization in clinical studies describing an HDI. The values for each of the lines were assigned after the first round. These values were discussed and adapted during the different runs to reach a consensus. Answers with a * automatically deactivate the subsequent questions

*N*°	Question	Points		
		Yes	No	NA
(A) Herbs				
1	The study concerns 3 herbs or more	NA*	0	/
2	The name of the herbal food supplement or herbal drug product is mentioned	2	NA*	/
3	The study concerns a single molecule usually present within a mixture mix in the herb	0	NA*	/
4	The name of the herb leaves no ambiguity or the binomial Latin name is specified	1	-1	/
5	The herb part used is mentioned	1	-1	/
6	The extraction solution is mentioned	1	-1	/
(B) Mechanism				
1	The study describes an interaction	0	4	/
2	The event is a pharmacodynamic event	5	0	/
3	The event is a pharmacokinetic event	3	0	/
4	The study describes the implication of a herb on one or multiple enzymes or transporters of which the drug is a substrate	2	1	0
5	The drug is a substrate for one or more enzymes or transporters	2	1	0
6	If the drug is a CYP2C9, 2C19 or 2D6 substrate, the patient’s genotype is described	-1	-2	0
7	The study concerns a single intake of the herb	3	2	0
8	The statistical analysis is coherent with the conclusions and the relevance of the test is clear	0	-2	0
(C) Cohort				
1	The cohort of the study includes less than 10 subjects	1	2	/
2	The cohort of the study includes more than 20 subjects	2	0	/
3	The study concerns a single ethnic group	-1	2	0
4	The composition of cohort is biased in terms of sex ratio or age groups	-1	2	0
5	The study concerns healthy volunteers	2	0	0
6	If the patients suffer from heart/kidney/liver failure, cancer, neurological disorders, immunosuppression, or are transplanted, the treatment implied in the event is directly related to this pathology	2	-2	0
7	A conflict of interest is identified (i.e. The manufacturer or seller of the herb is an author or co-author)	-1	0	0

The next criterion considers herb’s description. If a herb is referenced in an article by the name of a herbal food supplement that contains it (criterion n°2), we can be rather confident about the correctness of the description. Yet, herbal food supplements are not as strictly controlled as herbal medicinal products, and variations in dosages may occur, leading to potential differences between batches. An article corresponding to this case will be assigned a score of 2 for this section, which is 1 point below the maximum. If the study concerns a single molecule only (criterion n°3), the evidence is lower as the corresponding herb is a completely different matrix with complex molecules combination, which has the potential to lead to different clinical effects. For this reason, we decided to assign a 0 in this case. The three following criteria concern herbs as such. The first of these criteria ensures that herb’s binomial Latin name is specified (criterion n°4). Even though vernacular name could be sufficient in some cases, binomial Latin name ensures to avoid some confusions. For example, “ginseng” can refer among others to *Panax ginseng* C. A. Mey., *Panax quinquefolius* L. or *Panax notoginseng (Burkill)* F. H. Chen ex C. Y. Wu & K. M. Feng (Araliaceae), but also to unrelated species used as adaptogens. Another crucial piece of information is the part of the herb used (criterion n°5), that will qualitatively and/or quantitatively influence the chemical composition of the herb. The last criterion is the extraction method (criterion n°6), which will also greatly influence the final composition of the herbal product depending on physico-chemical properties of the herbal components. The last three criteria being interdependent in order to get a satisfying description, we decided to base their score on this dependence. A satisfied criterion will give 1 point, while a non-satisfied one will account for − 1. Thus, if all criteria are fulfilled, we get the highest possible score as we have the best degree of description available through an article, at least without diving into complex composition description that would require specific knowledge to interpret. Yet, if at least one of these criteria is not fulfilled, the score drastically drops. Noteworthily, refined specifications were not considered, regarding the profiles of the potential users of this scaling method. Other quality issues which can hardly be assessed (e.g. fraud) were not considered. Nevertheless, clinical studies hardly provide specifications or control steps.

#### Interaction mechanism

The “Mechanism” section considers pharmacological clues about the interaction. It aims to assess how the event can be expected to be linked to concomitant use of the herb and the drug given their known PK and PD behaviors. The first three criteria concern the nature of the event. The article can either describe the absence of clinical effect when the herb and the drug are given concomitantly (Table 1 (B) and Table 2 (B) criterion n°1), a PD event (Table 2 (B) and Table 2 (B) criterion n°2) or a PK event (Table 2 (B) and Table 1 (B) criterion n°3). If no event occurred as considered in criterion n°1, we can be reasonably confident about the interpretation of the study as there is no pharmacological characteristics of interaction to investigate. In this case, we decided to give the section a good score (4 points). In other cases, we decided to give 5 points for PD events, as they are usually easier to predict, and to give 3 points for PK events, which are much harder to interpret due to the number of impacting factors. The next criteria inquires whether the interaction implies a single or multiple enzymes or transporters (Table 1 (B) criterion n°4 Table 2 (B) criterion n°4 and n°5). We consider this criterion important to identify the enzymes or transporters involved in the interaction. Indeed, this is a crucial step in assessing the link between the products and the described event. If multiple pathways are known, it becomes much harder to determine the mechanism of interaction. Pharmacogenetics may also influence the interaction, which is another concern. In particular, the implication of CYP2C9, CYP2C19 and CYP2D6 can lead to large interindividual variations and thus is to be considered (Table 1 (B) criterion n°5 and Table 2 (B) criterion n°6). The implication of these isoenzymes leads to harder generalization of potential clinical events [16]. The chronology of the event is also a key factor to be considered to assess the potential interaction. An event occurring directly after a single intake of the herb is a greater clue than if it occurs after some days/weeks or after multiple intakes (Table 1 (B) criterion n°6 and Table 2 (B) criterion n°7). Yet, we cannot completely penalize prolonged periods of intakes, as some events are known to occur only after some days/weeks (for example, enzymatic inductions are known to take some weeks to take place) [17]. The presence of a dechallenge or rechallenge, i.e. an improvement when a member of the interaction is withdrawn and/or reappearance when it is reintroduced (Table 1 (B) criterion n°7) is also a great clue of implication of the drug/herb considered in the event, though this information is rarely available. Finally, for clinical studies, the proper statistical analysis, the relevance of tests used and statistical significance must be considered (Table 2 (B) criterion n°8).

#### Patient or cohort key information

An important section concerns the characteristics of the patients, defined in two separate tables: a table for a single patient in the case of a case report (or case series) (Table 1 (C)), and a table for the cohorts of clinical studies (Table 2 (C)). Concerning patients, the first characteristic to be considered is the age of patients (Table 1 (C) criterion n°1 and Table 2 (C) criterion n°4). Given that children and elderly patients might answer differently to the same herb/drug combination compared to adults, this information should be considered when trying to infer the risk in general population. A direct consequence of this is the health status and specific conditions of patients (Table 1 (C) criteria n°1 and n°2 and Table 2 (C) criteria n°5 and n°6). Two cases are to be distinguished here: the implied treatment can be related to the condition (i.e. an interaction involving immunosuppressants in a transplanted patient) or not (i.e. an interaction involving an anti-platelet drug in a transplanted patient). In the first case, the interaction is worth noting as we can expect other patients with the same condition to suffer from the same events and thus increase our vigilance about this interaction. In the second case, a potential event might be partially due to the specific condition of the patient and be less informative for general population. Even though this case is still informative in specific situations that should not be overlooked, we considered it less relevant when related with our goal of defining a generalized scale. In case reports, we also wanted to ensure that patient’s treatment was well balanced before introduction of the herb (Table 1 (C) criterion n°3). If it is not the case, defining whether the event is linked to the treatment or to an interaction is more hazardous. In the case of clinical studies, a crucial factor is the cohort composition. This includes the size of the cohorts (Table 2 (C) criteria n°1 and n°2), ethnicity (Table 2 (C) criterion n°3), sex and age (Table 2 (C) criterion n°4) and health status (Table 2 (C) criteria n°5 and n°6).

#### Proper use

The last criterion we decided to include to establish event causality is the proper use of the herb. We scanned the literature about those events and especially in case reports and we found out that herbal products are often consumed in unpredictable ways. For example, there are cases of patients suffering from adverse events while consuming two liters of St John’s wort (*Hypericum perforatum* L.) tea a day [18] or death potentially related to recreational use of kratom (*Mitragyna speciosa* Korth.) [19]. To distinguish cases in which herbal products are used as recommended by manufacturers or guidelines from those in which they are not, we defined general criteria for different herbal preparations. When the use does not respect recommendations, the score is reduced by 2 points. If usage is not described, it is reduced by 1 point, considering the lack of information while still not scoring it as abusive. In other cases, one point is added to the score.

### Assessing warning level of an event

Given the scores defined above, we can now assign a warning level to the event. This warning level should consider reliability of the case described as well as the severity of the event and its propensity to be extrapolated in general population. To take both severity and reliability into account, we decided to use a matrix to generate the final warning level. This matrix is composed of the severity of the event (Fig. 1) on the first dimension and of the reliability level on the second dimension (Tables 1 and 2). The reliability level is composed of the scores of all sections (Table 1 (A), (B), (C) and (D), Table 2 (A), (B) and (C)) synthesized in a unique score (Table 3). The final matrix is shown in Table 4.

**Table 3:** Thresholds for sections synthesized scores. These scores are obtained by adding the quotations obtained for each of the lines and 3 brackets have been designed to define whether we consider the descriptions to be good, just correct (average) or poor

Value	1 (Poor)	2 (Average)	3 (Good)
Clinical study			
Herb	< 0	0–1	> 2
Mechanism	< 2	2	> 2
Patient	<-2	-2–3	> 3
Use	< 0	0–1	> 2
Case report			
Herb	< 0	0–1	> 2
Mechanism	< 0	0–3	> 3
Cohort	< 0	0–3	> 3

**Table 4:** Final risk assessment score matrix

	Poor	Average	Good
Case report			
Grade 0	No risk known	No risk known	No risk known
Grade 1	No risk known	No risk known	Unsignificant interaction
Grade 2	No risk known	No risk known	Unsignificant interaction
Grade 3	Unsignificant interaction	Unsignificant interaction	Expert interpretation required
Grade 4	Expert interpretation required	Expert interpretation required	Expert interpretation required
Grade 5	Expert interpretation required	High risk	High risk
Grade 6	Expert interpretation required	High risk	High risk
Clinical study			
Grade 0	No risk known	No risk known	No risk known
Grade 1	No risk known	Unsignificant interaction	Unsignificant interaction
Grade 2	No risk known	Unsignificant interaction	Unsignificant interaction
Grade 3	Unsignificant interaction	Expert interpretation required	Expert interpretation required
Grade 4	Expert interpretation required	Expert interpretation required	Expert interpretation required
Grade 5	Expert interpretation required	High risk	High risk
Grade 6	Expert interpretation required	High risk	High risk

Using the scores obtained in Table 4, we have summarized the results obtained from the severity scores AND the imputability/generalization scores. These scores are presented in Table 4.

The final reliability level is defined by synthesizing scores of all sections. The synthesized score ranges from 1 (bad) to 3 (good) and represents the quality of description of the given item in the article. Each of these scores is based on thresholds shown in Table 3. These thresholds are arbitrary and based on the minimum and maximum possible scores obtainable in each section. Final reliability index is obtained by averaging synthesized scores for all sections and rounding it down. By using a simple arithmetic mean, we give the same importance to all sections and thus penalize articles in which any section is poorly described. This choice was motivated by our belief that any event in which one of these sections lacks information greatly reduces confidence in its conclusion, whatever the concerned section.

These matrices can be accessed directly from the website. To simplify the scorer’s interpretation, a color code is used to help the user to make a quick decision.

## Discussion

In contrast with ADR implying drug-drug interactions, published data describing HDI are heterogeneous, with large disparity in descriptions. This heterogeneity concerns in particular herbal products description including proper naming of the herb and herb part used, extract preparation, dosage, etc. Besides, these data sometimes lack precision, especially in case reports as they are based on patient’s speech and on clinical description from health practitioners who are often inexperienced with these issues. Existing tools that handle HDI also lack homogeneity and ease to interpret due to the lack of unified vocabulary to express the risk. This results in a description based on experts’ intuition and lack of transparency and robustness.

To structure HDI description, we propose a unified scaling process. We did this by following the broad lines of the Delphi process, consulting several experts in the field (in the early rounds) or people whose background could lead them to use BABINE. The Delphi process allowed us to design a pertinent and appropriate scale by collecting and refining the experience of a panel of experts [20]. The design process of an HDI risk scale requires a certain number of approximations and concessions. The different iterations with experts and scorers have brought that to light. At the end of the process, we managed to reach a consensus indicated by a small dispersion of the expert’s answers. At each step, dispersion between expert’s answers were evaluated, and they were contacted in case of question about what lead to a specific answer. From these discussions, two major sources of discordance were identified:

- Misunderstanding of the question.
- Missed information while reading the published peer-reviewed publications.

To reach this consensus throughout iterations, we thus had to refine questions step by step so that everyone grasped the meaning of the criteria. Multiple strategies have been used to reach better clarity, including redefining some specific terms or changing questions formulation. In some cases, it was not possible to reach a consensus because of an interpretative component of the question. This is for example the case in questions implying the existence of bias, which is impossible to define exactly for every single situation. In these cases, scores associated with the criterion were reduced to offset this interpretative component. The second factor is much harder to handle as filling in the form takes some time and finding a specific piece of information in a clinical study requires attention. Number of questions are divisive, even when they are interpreted similarly by experts. One example of these discordance concerns the severity grade of the interaction. One of the articles used for construction of the method describes the case of a HIV positive man showing an increased viral load without any clinical symptoms after *Ginkgo biloba* L. consumption [21]. Expert’s answers were then divided between Grade 1 indicating no clinical symptom or Grade 4 or 5, indicating a risk linked to loss of clinical effectiveness in a serious disease. The two points of view are defensible, and clarifying this ambiguity seems impossible while keeping the scale simplicity. Tools such as CTCAE, which include hundreds of tables to precisely describe any type of clinical manifestations, reinforce this belief.

Security [22] and help mechanisms are also included on the online version of the scale in BABINE. At first, a guided tour introduces the scale to the user. On incompatible answers combination, a warning is proposed. For example, if the user declares an interaction but says that the interaction is neither pharmacokinetic nor pharmacodynamic, a warning popup will appear. Finally, tooltips present under some questions which could be ambiguous give information about the thinking behind the criteria.

We are aware that BABINE process contains approximations and is sensitive to human interpretations. A possible future prospect would be to provide artificial intelligence all criteria to score more mechanically/automatically in an unbiased manner and use text mining to help users find relevant information in the text. We plan to test this in the future.

## Conclusions

Handling HDI is not an easy task. It requires deep knowledge of pharmacognosy/phytochemistry and pharmacology/toxicology, including extensive knowledge of drug metabolization pathways. Besides the required knowledge, interindividual variations still increase difficulty to predict such HDI. Yet, even if it cannot replace professional advice, it is important to be able to rationalize this interpretation based on objective criteria, for example when textually describing an HDI such as in articles or databases.

If scientific literature provides validated data, few tools exists to interpret raw clinical data presented in these studies. Our goal for this work is to define such a tool in the form of an evaluation scale. Our objective is to make this scale user friendly while still able to generalize most of the descriptions of interactions encountered in literature. In collaboration with a panel of pharmacologists, medical doctors, pharmacovigilants, pharmacognosts and physicians, we defined a scale based on two aspects of pharmacological events: severity of the observed reaction and attributable causality of this event to described products combination.

After rounds of assessment and adaptation, we believe that this tool could reach our goals as we obtained relatively comparable evaluations of events described in different articles. Answers to defined items, if not always strictly similar, were most of the time close and provided similar risk estimation in the end. Yet, some sections have shown to be more prone to divergence. This is the case for sections concerning cohort, interaction mechanism or herb definition. Besides, clinical studies are much harder to interpret due to the diversity and profusion of parameters involved as well as time and concentration required to read them. This is why we have designed a user-friendly flowchart.

Even though we chose to trade some precision against usability, we believe that this tool allows to define an attention grade for HDI that is sufficiently transparent and robust for general phytovigilance applications.

## Electronic supplementary material

Below is the link to the electronic supplementary material.

Supplementary Material 1

## Data Availability

No datasets were generated or analysed during the current study.

## Declarations

No experiment has been done in this work on humans and/or the use of human tissue samples.
