# Supplementary material for: BABINE: An original and user-friendly scale for the simple and quick management of herb-drug interactions in clinical practice
Source: BMC Complement Med Ther. 2024 Dec 18;24:414. doi: 10.1186/s12906-024-04706-9 (PMC11654206; doi:10.1186/s12906-024-04706-9)
Supplement: Supplementary file 1 — Supplementary Material 1 [file 12906_2024_4706_MOESM1_ESM.docx]

BABINE: An original scale for management of herb-drug interactions in clinical practice

Anthony Cnudde^1,2^, Camille Allely^3^, Natacha Biset^1^, Pierre Champy^4^, Nathalie Fouilhé^5^, Fanny Huret^6^, Sibi Lawson^7^, Aline Mercan^8^, Doris Pascale Noukela Noumi^7^, Serge Michalet^10^, Andrea Montis^9^, Stephanie Pochet^1^, Audrey Schils^1^, Cecilia Tangeten^9^, Michel Tod^8^, Pierre Van Antwerpen^9^, Audrey Vervacke^11^ and Florence Souard^1*^

^1^ Department of Pharmacotherapy and Pharmaceutics, Faculty of Pharmacy, Université libre de Bruxelles (ULB), Brussels, Belgium.

^2^ Machine Learning Group, Faculty of Sciences, Université libre de Bruxelles (ULB), Brussels, Belgium.

^3^ CNHIM, Theriaque, France.

^4^ Équipe Chimie des Substances Naturelles, BioCIS, CNRS, Université Paris-Saclay, Orsay, France.

^5^ Centre Hospitalier Universitaire Grenoble Alpes, Grenoble, France.

^6^ Nutrition Risk Assessment Unit, French Agency for Food, Environmental and Occupational, Health & Safety (ANSES), Maisons-Alfort, France.

^7^ Université Libre de Bruxelles, Brussels, Belgium.

^8^ Hôpital de la Croix-Rousse, Hospices Civils de Lyon, and LBBE, Université Claude
Bernard Lyon 1, Lyon, France.

^9^ RD3 Unit of Pharmacognosy, Bioanalysis and Drug Discovery, Faculty of Pharmacy, Campus Plaine, Université Libre de Bruxelles, 1050 Brussels, Belgium

^10^ Pharmacognosy, Department of Drug Sciences, Institut des Sciences Pharmaceutiques et Biologiques (ISPB) / UMR 5557 Ecologie Microbienne, Université Claude Bernard Lyon 1, Lyon, France.

^11^ Hôpital universitaire de Bruxelles - site Erasme (HUB) BE

Supporting information with all phases of case reports and clinical study forms (Tables S1 to S8 and calculated entropies (Figure S1 to S10) to examine discrepancies and improve the questionnaire towards a next version.

Case reports form – phase 1

Table S1: First version of the form for case reports scoring

|  | |  | | |
| --- | --- | --- | --- | --- |
| **N°** | **Item** | **Yes** | **No** | **NA / Unknown** |
| Herb (H) | | | | |
| H1 | The case concerns 3 herbs or more or the patient takes more than 3 drugs | The case is not interpretable |  |  |
| H2 | The name of the complement is mentioned | +2 | 0 | / |
| H3 | The case concerns a single molecule | +1 | 0 | / |
| H4 | If the name of the complement is not mentioned: Latin name of the herb is mentioned or leaves no ambiguity | +1 | -1 | / |
| H5 | If the name of the complement is not mentioned: the part of the herb used is mentioned | +1 | -1 | / |
| H6 | If the name of the complement is not mentioned: the extraction solution is mentioned | +1 | -1 | / |
| Mechanism | | | | |
| M1 | The mechanism of interaction is pharmacodynamic | +3 | 0 | 0 |
| M2 | The interaction is pharmacokinetic (enzymatic induction or inhibition) for the herb and the drug is substrate of a single CYP | +2 | -2 | 0 |
| M3 | The metabolization involves CYP 2B6/2C9/2C19 | -1 | +1 | 0 |
| M4 | The case concerns a single intake of the herb | +3 | 0 | 0 |
| M5 | The case concerns multiple intake of the herb | +2 | 0 | 0 |
| M6 | A rechallenge/dechallenge has been observed | +3 | 0 | 0 |
| M7 | The suspected mechanism is enzyme inhibition and the plant was taken repeatedly over 7 days. | +2 | 0 | 0 |
| Patient | | | | |
| P1 | The case concerns a patient aged 70 or more, 18 or less, or a pregnant woman | -2 | 0 | -2 |
| P2 | The treatment is directly related to the pathology of a patient with a transplant, heart/kidney/liver failure, cancer or neurological disorders or immunosuppressed | +2 | -2 | 0 |
| P3 | The patient’s treatment was well balanced before introduction of the herb | +2 | 0 | / |

### Respondents’ agreement scores

Each participant independently scored 2 publications (case reports) describing an interaction between 1 plant and 1 drug:

1. Chan, J.C.M.; Ng, M.-H.; Wong, R.S.M.; Tomlinson, B. A Case of Simvastatin-Induced Myopathy with SLCO1B1 Genetic Predisposition and Co-Ingestion of Linagliptin and Stevia Rebaudiana. *J. Clin. Pharm. Ther.* **2019**, *44*, 381–383, doi:10.1111/jcpt.12805.
2. Naccarato, M.; Yoong, D.; Gough, K. A Potential Drug-Herbal Interaction between Ginkgo Biloba and Efavirenz. *J. Int. Assoc. Physicians AIDS Care Chic. Ill 2002* **2012**, *11*, 98–100, doi:10.1177/1545109711435364.

The results were compared and analyzed. Entropy was calculated (Figure S1) to examine discrepancies and improve the questionnaire towards a second version.

### Discussions for the first phase of evaluation:


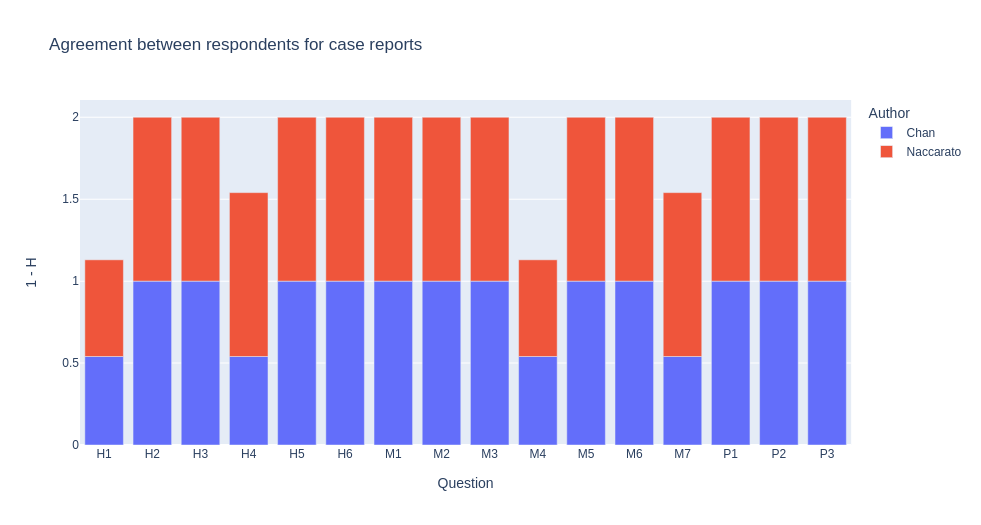
Figure S1: Agreement between respondents for two case reports in first phase. Agreement was computed as entropy value: $H=-\sum_{i} p_{i}*{log}_{4}\left( p_{i} \right)$. For clarity purpose, results are presented as 1 - H to assign high values to low entropy. A higher value means a better agreement between respondents; for a single article, a value of 1 means that every respondent gave the same answer while a value of 0 means that they all gave different answers. N=4.

The discrepancies concerned questions H1, H4, M4 and M7.

- H1: disagreement was due to a lack of attention after discussion during a focus group. In Chan publication, a sentence stated ‘herbal supplements which included oral stevia [*Stevia rebaudiana* Bertoni]’ , which indicates that multiple herbal products were involved. This information was missed by one of the respondents. The same thing happened in Naccarato study where flax oil, rutin, Ginkgo biloba and horse chestnut are mentioned. Note that for testing purposes, even if in a typical use case the article should have been discarded from analysis due to the outcome of this question, we decided to analyze the rest of the form to gather information about its robustness.
- H4: In Chan’s case report, the Latin name is given for Stevia, but not horse chestnut. Some participants answered yes due to the fact that stevia is primarily focused by the authors, while one of the respondants answered ‘no’ because of the horse chestnut. This question could be adapted to reduce ambiguity, but no unambiguous solution has been found.
- M4 and M7: Both questions lead to disagreement due to lack of temporal information on herb intake. In Chan, few information about temporality is given to answer M7 (‘Additionally, he was also consuming multiple vitamins and herbal supplements which included oral stevia [*Stevia rebaudiana* Bertoni] for 2–3 months.’). M4 is ambiguous: when the case concerns multiple intakes, should one answer ‘no’ or ‘NA’? This does not change the score as both answers lead to +0, but this can be semantically misleading.

Case reports form – phase 2

During the first focus group, all the questions were reviewed with the participants and any changes are shown in red.

Table S2: Second version of the form for case reports scoring

|  | |  | | |
| --- | --- | --- | --- | --- |
| **N°** | **Item** | **Yes** | **No** | **NA / Unknown** |
| Herb | | | | |
| H1 | The study concerns 3 herbs or more ~~or the patient takes more than 3 drugs~~ | / | 0 | 0 |
| H2 | The name of the complement/drug is mentioned | +2 | 0 | 0 |
| H3 | The case concerns a single molecule | +1 | 0 | 0 |
| H4 | ~~If the name of the complement is not mentioned :~~The binomial Latin name of the herb is specified or ~~leaves no ambiguity~~ | +1 | -1 | 0 |
| H5 | ~~If the name of the complement is not mentioned :~~The herb part used is mentioned | +1 | -1 | 0 |
| H6 | ~~If the name of the complement is not mentioned :~~The extraction solution is mentioned | +1 | -1 | 0 |
| Mechanism | | | | |
| M1 | The interaction mechanism described is pharmacodynamic | +3 | 0 | 0 |
| M2 | ~~The interaction is pharmacokinetic (enzymatic induction or inhibition) for the herb and the drug is substrate of a single CYP~~  The study describes the implication of a single enzyme or transporter (herb) and the drug is substrate of this enzyme or transporter only – major pathway (refer to HUG substrates table or DDI predictor) | +2 | -2 | 0 |
| M3 | The drug is metabolized by the CYP2C9, 2C19 or 2D6 | -1 | +1 | 0 |
| M4 | The study describes an event that appeared after a single intake of the herb | +3 | 0 | 0 |
| M5 | ~~The study describes an event that appeared after multiple intake of the herb~~ The number of intakes of the herb is: | Single intake: +3 | Multiple intakes: +2 | 0 |
| M6 | If and only if an interaction occurred: a dechallenge or rechallenge was stated | +3 | 0 | 0 |
|  | ~~The mechanism described is an enzymatic inhibition and the herb has been taken repeatedly over at least 7 days.~~ | ~~+2~~ | ~~0~~ | ~~0~~ |
| Patient | | | | |
| P1 | The case concerns a patient aged 70 or more, 18 or less, or a pregnant woman | -2 | 0 | -2 |
| P2 | If the patient has been transplanted, has a heart/kidney/liver failure, cancer or neurological disorders or is immunosuppressed, the treatment is directly related to this pathology | +2 | -2 | 0 |
| P3 | The patient’s treatment was well balanced before introduction of the herb | +2 | 0 | 0 |
| Good use | | | | |
| G1 | If and only if the interaction is due to an herbal tea: the patient consumes more than 1 liter of herbal tea per day (if it is not an herbal tea, mark it as unknown) | -2 | +1 | -1 |
| G2 | If and only if the interaction is due to a dietary supplement or powder: the patient has exceeded the dosage indicated by the manufacturer (if it is not a dietary supplement or powder, mark it as unknown) | -2 | +1 | -1 |
| G3 | If and only if the interaction is due to a fruit juice: the patient has consumed more than 500 mL of juice per day (if it is not a juice, mark it as unknown) | -2 | +1 | -1 |

## Modifications:

1. H1: Mentions of drugs in this question were removed as the section focuses on description of the herb. As usage of drugs are usually better monitored than herbs, we considered here that the presence of multiple drugs causes less ambiguity than mix of herbs.
2. H4, H5, H5: To clarify questions, the first part of the sentence was removed and the questions where deactivated in the form when ‘yes’ was answered for H2 or H3 to prevent answers. This change responds to case in which users answer to H4, H5 and H6 even though they answered ‘yes’ for H2 or H3.
3. M2 was changed for clarity purposes. The questions now clearly ask for implication of an enzyme or transporter.
4. The question ‘The mechanism described is an enzymatic inhibition and the herb has been taken repeatedly over at least 7 days’ has been removed. This question was found to be confusing due to the objective number of days stated. Besides, it requires more information than usually present in case reports to be useful.
5. P2: “IF” has been added at the beginning of the sentence to emphasizes the fact that this question makes sense only if the patient actually suffers from one of cited pathologies. This was actually not the case for all respondents.
6. G1-3: An item about how the herbal product was used by the patient was missing. These questions should help to distinguish case in which a product was consumed excessively (e.g. a woman drinking 2 L of St John’s Wort tea per day, what cannot be extended to all use cases)

### Discussions for the second phase of evaluation:

Discussion with experts lead new criteria, especially about G1-3 that are missing lots of herbal products. It was decided to improve the form before testing it.

Case reports form – phase 3

Table S3: Third version of the form for case reports scoring

|  | |  | | |
| --- | --- | --- | --- | --- |
| **N°** | **Item** | **Yes** | **No** | **NA / Unknown** |
| Herb | | | | |
| H1 | The case concerns 3 herbs or more | / | 0 | 0 |
| H2 | The name of the supplement or phytomedicine is mentioned | +2 | 0 | 0 |
| H3 | The study concerns a single molecule | +1 | 0 | 0 |
| H4 | The binomial Latin name of the herb is specified or leaves no ambiguity | +1 | -1 | 0 |
| H5 | The herb part used is mentioned | +1 | -1 | 0 |
| H6 | The extraction solution is mentioned | +1 | -1 | 0 |
| Mechanism | | | | |
| M1 | The case describes an interaction (if the case describes the absence of interaction, select 'no') | 0 | +4 | / |
| M2 | The interaction mechanism described is pharmacodynamic | +5 | 0 | / |
| M3 | The interaction mechanism described is pharmacokinetic | +3 | 0 | / |
| M4 | The study describes the implication of a single enzyme or transporter (herb) and the drug is substrate of this enzyme or transporter only – major pathway (refer to HUG substrates table or DDI predictor) | +2 | -2 | 0 |
| M5 | The drug metabolization involves the CYP2C9, 2C19 or 2D6 | -1 | +1 | 0 |
| M6 | The study describes an event that appeared after ~~a single intake of the herb~~ | A single intake of the herb:  +3 | Multiple intake of the herb:  +2 | 0 |
|  | ~~The study describes an event that appeared after multiple intake of the herb~~ | ~~+2~~ | ~~0~~ | ~~0~~ |
| M7 | ~~If and only if an interaction occurred:~~ A dechallenge or rechallenge was observed | +3 | 0 | 0 |
| Patient | | | | |
| P1 | The case concerns a patient aged 70 or more, 18 or less, or a pregnant woman | -2 | 0 | -2 |
| P2 | If the patient has been transplanted, has a heart/kidney/liver failure, cancer or neurological disorders or is immunosuppressed, the treatment is directly related to this pathology | +2 | -2 | 0 |
| P3 | The patient’s treatment was well balanced before introduction of the herb | +2 | 0 | 0 |
| Good use – Only **one** of these questions should be answered | | | | |
| G1 - 1 | ~~If and only if the interaction is due to a dietary supplement or powder:~~ Supplement: T~~he patient has exceeded the dosage indicated by the manufacturer~~ | -2 | +1 | -1 |
| G1 - 2 | ~~If and only if the interaction is due to an herbal tea:~~ Tea: The patient consumes more than 1 liter of herbal tea per day ~~(if it is not an herbal tea, mark it as unknown)~~ | -2 | +1 | -1 |
| G1 - 3 | ~~If and only if the interaction is due to a fruit juice:~~ Juice: The patient has consumed more than 500 mL of juice per day ~~(if it is not a juice, mark it as unknown)~~ | -2 | +1 | -1 |
| G1 - 4 | Powder: The patient took more than one teaspoon a day | -2 | +1 | -1 |
| G1 - 5 | Essential oil: The patient took more than 10 drops a day | -2 | +1 | -1 |
| G1 - 6 | Recreational use: The case concerns a recreational use | -2 | +1 | -1 |
| G1 - 7 | Other: The patient did not respect manufacturer recommendations | -2 | +1 | -1 |

### Modifications for this round

1. H2: The term ‘drug’ has been changed for ‘phytomedicine’ to avoid confusions. The term drug was used to describe herbal products with the legal status of ‘drug’ but was not understood this way by all respondents.
2. M1: This question was added to distinguish the particular case in which no interaction occurred, what simplifies the rest of the section. In the tool provided to users to score the article, answering ‘no’ to this question leads to deactivation of the rest of the section.
3. M6: two questions were fused as one to reduce mental workload of the form.
4. M7: Removed first part of the sentence, as the question should not be answered when no interaction occurs anyway (as described in M1)
5. Good use: A set of questions has been added to cover a broader spectrum of herbal products. One question should be answered at a time. In the tool provided to users to score the article, the question is selected from a dropdown, avoiding multiple answers.

### Respondents agreement scores after modifications – Experts

Each participant independently scored 2 others publications (case reports) describing an interaction between 1 herb and 1 drug:

1. Lee, A.; Chui, P.T.; Aun, C.S.T.; Gin, T.; Lau, A.S.C. Possible Interaction between Sevoflurane and Aloe Vera. *Ann. Pharmacother.* **2004**, *38*, 1651–1654, doi:10.1345/aph.1E098.
2. Nayeri, A.; Wu, S.; Adams, E.; Tanner, C.; Meshman, J.; Saini, I.; Reid, W. Acute Calcineurin Inhibitor Nephrotoxicity Secondary to Turmeric Intake: A Case Report. *Transplant. Proc.* **2017**, *49*, 198–200, doi:10.1016/j.transproceed.2016.11.029.

The results were compared and analyzed. Entropy was calculated (Figure S2) to examine discrepancies and improve the questionnaire towards a second version.


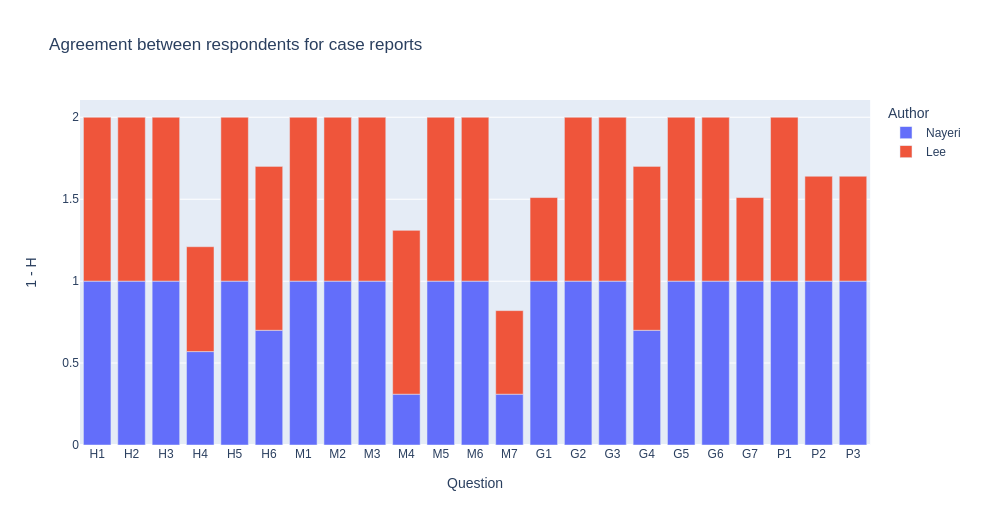
Figure S2: Agreement between respondents for two case reports in second phase. Agreement was computed as entropy value : $H=-\sum_{i} p_{i}*{log}_{i}\left( p_{i} \right)$, with i the number or possible answers for the question. For clarity purpose, results are presented as 1 - H to assign high values to low entropy. A higher value means a better agreement between respondents; for a single article, a value of 1 means that every respondents gave the same answer while a value of 0 means that they all gave different answers. N=11.

### Discussions for the third phase of evaluation - Experts:

This version of the questionnaire was tested during 2 rounds. The first time with the co-authors of this article and a second time with master's students from the ULB pharmacy faculty. The results of the 2 rounds are detailed below. Article used is:

1. Chan, J.C.M.; Ng, M.-H.; Wong, R.S.M.; Tomlinson, B. A Case of Simvastatin-Induced Myopathy with SLCO1B1 Genetic Predisposition and Co-Ingestion of Linagliptin and Stevia Rebaudiana. *J. Clin. Pharm. Ther.* **2019**, *44*, 381–383, doi:10.1111/jcpt.12805.

- H4: The part ‘leaves no ambiguity’ is problematic as it leads to difference between pharmacognosts/phyto-vigilance experts and other experts, as the former are more demanding about herb denomination (species, subspecies, variety, ...). Yet, removing this part can in turn lead to way to strict evaluation when there is no doubt about herb used but when no Latin name is stated.
- H6: As for H4, the difference of background of experts leads to difference in interpretation for the same reasons.
- M4: The question is hard to grasp and still leads to disagreement. Some divergent answers appear, but after discussion, no unambiguous way to simplify the question could be found.
- G questions: Two sources of divergence appear here: the type of product used, and the potentially problematic consumption by the patient. Here, supplement, powder and other were selected. This confusion is not a problem as they all lead to same scores when the same answer is given, the differentiation between different product is only there to define the threshold for the ‘acceptable use’. The second source of confusion coming from acceptable use is harder to assess. This is due to the fact that defining what is exaggerated when speaking about herbal product is not straightforward, some experts being again more strict than other. Overall, the entropy remains quite low, and the disagreement is due to only three experts out of 11.
- P2 – P3: Confusion here was due to the nature of the treatment. In this case, the drug involved was sevoflurane, an anesthetic, and was thus a one-off. Disagreement for P2 is due to some experts over-interpreting the operation as part of pathologies cited in the question. Disagreement in P3 is due to confusion between the one-off and long-term treatments usually seen in case reports. Experts with divergent answers (a minority, as shown by the low entropy) agreed that the question was clear and that the P3 item in not applicable here.

### Respondents agreement scores after modifications – Master students

### Discussions for the third phase of evaluation – Students:


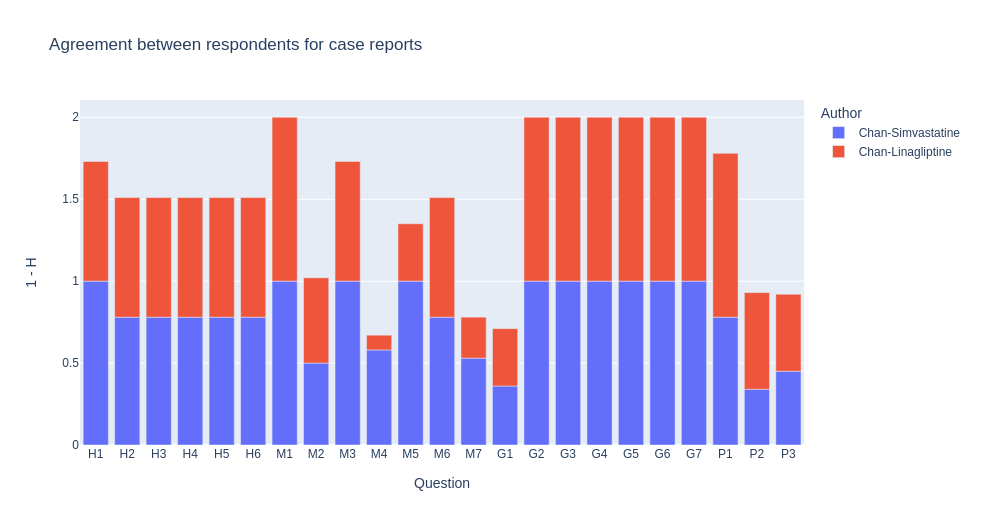
Figure S3: Agreement between respondents for two case reports in third phase. Agreement was computed as entropy value: $H=-\sum_{i} p_{i}*{log}_{i}\left( p_{i} \right)$, with i the number or possible answers for the question. For clarity purpose, results are presented as 1 - H to assign high values to low entropy. A higher value means a better agreement between respondents; for a single article, a value of 1 means that every respondents gave the same answer while a value of 0 means that they all gave different answers. N=11 for Chan-Simvastatine, N=8 for Chan-Linagliptine

1. Results for students (second Master) are overall worse than experts. This is natural and can be specifically identified in technic questions:
   1. M2-M4-M7: There is a lack of understanding of pharmacology: difference between PK and PD mechanisms, dechallenge/rechallenge, metabolization pathways, …
   2. M4: The question also causes problems to students, as it could be expected.
2. P2: This item was sometimes problematic for some experts, due to the ‘condition’ present in the question. Yet, this article previously proposed to experts (phase I) did not cause any specific issue, and after discussion, it could be due to a lack of knowledge/comprehension of students.
3. P3: Again, it did not cause too much issues for experts, and the difference seems to be due to the understanding of ‘balanced treatment’.
4. In general, students had a limited time to fill the forms due to schedule, and hurry was also cited as an error factor.
5. Case reports form – phase 4
6. For the last round, only minor changes were necessary. At this stage, the major advances have been visual, with the addition of pop-ups in particular. A new pair of case reports was used alongside previous ones 5.
7. Table S4: Fourth version of the form for case reports scoring

|  | |  | | |
| --- | --- | --- | --- | --- |
| 1. **N°** | 1. **Item** | 1. **Yes** | 1. **No** | 1. **NA / Unknown** |
| 1. Herb | | | | |
| 1. H1 | 1. The study concerns 3 herbs or more | 1. / | 1. 0 | 1. 0 |
| 1. H2 | 1. The name of the complement/drug is mentioned | 1. +2 | 1. 0 | 1. 0 |
| 1. H3 | 1. The study concerns a single molecule | 1. +1 | 1. 0 | 1. 0 |
| 1. H4 | 1. The binomial Latin name of the herb is specified or leaves no ambiguity | 1. +1 | 1. -1 | 1. 0 |
| 1. H5 | 1. The herb part used is mentioned | 1. +1 | 1. -1 | 1. 0 |
| 1. H6 | 1. The extraction solution is mentioned | 1. +1 | 1. -1 | 1. 0 |
| 1. Mechanism | | | | |
| 1. M1 | 1. The case describes an interaction (if the case describes the absence of interaction, select 'no') | 1. 0 | 1. +4 | 1. / |
| 1. M2 | 1. The interaction mechanism described is pharmacodynamic | 1. +5 | 1. 0 | 1. / |
| 1. M3 | 1. The interaction mechanism described is pharmacokinetic | 1. +3 | 1. 0 | 1. / |
| 1. M4 | 1. The study describes the implication of a single enzyme or transporter ~~(herb)~~ and the drug is substrate of this enzyme or transporter only – major pathway (refer to HUG substrates table or DDI predictor) | 1. +2 | 1. -2 | 1. 0 |
| 1. M5 | 1. The drug metabolization involves the CYP2C9, 2C19 or 2D6 | 1. -1 | 1. +1 | 1. 0 |
| 1. M6 | 1. The study describes an event that appeared after a single intake of the herb | 1. +3 | 1. +2 | 1. 0 |
| 1. M7 | 1. A dechallenge or rechallenge was observed | 1. +3 | 1. 0 | 1. 0 |
| 1. Patient | | | | |
| 1. P1 | 1. The case concerns a patient aged 70 or more, 18 or less, or a pregnant woman | 1. -2 | 1. 0 | 1. -2 |
| 1. P2 | 1. If the patient has been transplanted, has a heart/kidney/liver failure, cancer or neurological disorders or is immunosuppressed, the treatment is directly related to this pathology | 1. +2 | 1. -2 | 1. 0 |
| 1. P3 | 1. The patient’s treatment was well balanced before introduction of the herb | 1. +2 | 1. 0 | 1. 0 |
| 1. Good use | | | | |
| 1. G1 - 1 | 1. Tea: The patient consumes more than 1 liter of herbal tea per day | 1. -2 | 1. +1 | 1. -1 |
| 1. G1 - 2 | 1. Supplement: The patient has exceeded the dosage indicated by the manufacturer | 1. -2 | 1. +1 | 1. -1 |
| 1. G1 - 3 | 1. Juice: The patient has consumed more than 500 mL of juice per day | 1. -2 | 1. +1 | 1. -1 |
| 1. G1 - 4 | 1. Essential oil: The patient took more than 10 drops a day | 1. -2 | 1. +1 | 1. -1 |
| 1. G1 - 5 | 1. Powder: The patient took more than one teaspoon a day | 1. -2 | 1. +1 | 1. -1 |
| 1. G1 - 6 | 1. Recreational use: The case concerns a recreational use | 1. -2 | 1. +1 | 1. -1 |
| 1. G1 - 7 | 1. Other: The patient did not respect manufacturer recommendations | 1. -2 | 1. +1 | 1. -1 |

### Modifications for this round

1. Only minor changes were done to case reports form for this round. Article used is:
2. Chan, J.C.M.; Ng, M.-H.; Wong, R.S.M.; Tomlinson, B. A Case of Simvastatin-Induced Myopathy with SLCO1B1 Genetic Predisposition and Co-Ingestion of Linagliptin and Stevia Rebaudiana. *J. Clin. Pharm. Ther.* **2019**, *44*, 381–383, doi:10.1111/jcpt.12805.
3. M4: The term ‘herb’ has been removed from the question to make question clearer as it does not bring more information to the question and was not understood by all respondents.

### Respondents agreement scores after modifications – Students

### Discussions for the fourth phase of evaluation – Students:


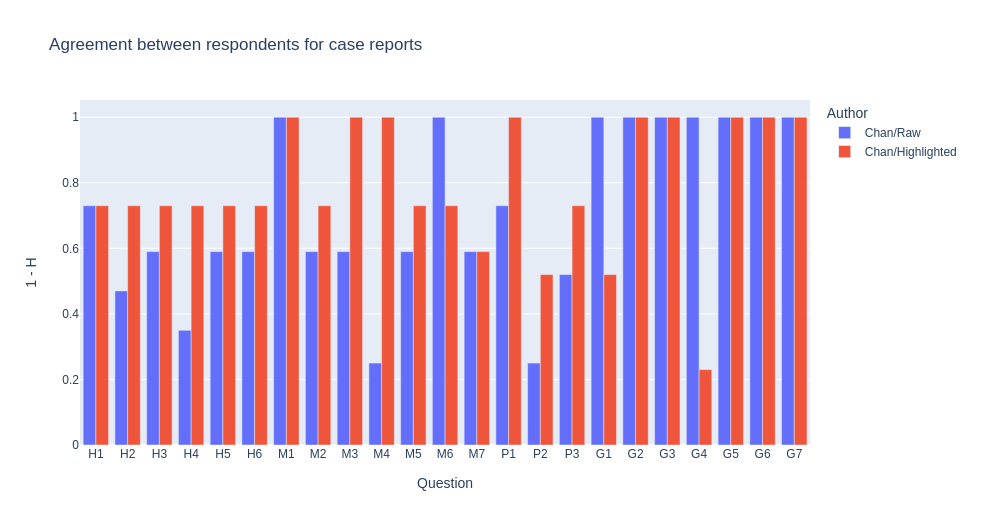
Figure S4: Agreement between respondents for 2 case reports in fourth phase - students. Agreement was computed as entropy value: $H=-\sum_{i} p_{i}*{log}_{i}\left( p_{i} \right)$, with i the number or possible answers for the question. For clarity purpose, results are presented as 1 - H to assign high values to low entropy. A higher value means a better agreement between respondents; for a single article, a value of 1 means that every respondents gave the same answer while a value of 0 means that they all gave different answers. The bars in red represents answers given by group for whom important information in the article were highlighted while bars in blue represents answers for non-highlighted text. N=17.

1. During this phase, we decided to test the impact of focus on the score. We already noted that filling the form takes time and require the user to find some specific pieces of information. Besides understanding of the question, one important factor that can lead to wrong answers is the lack of focus, people reading the article diagonally, or missing some “hidden” information (numbers in tables, information in footnotes, …). To try to objectify this effect, we divided a pool of students in two groups: one group received highlighted article while the other group received the same article without any annotation. The article selected presents two case reports:
2. Cordova, E.; Morganti, L.; Rodriguez, C. Possible Drug-Herb Interaction between Herbal Supplement Containing Horsetail ( Equisetum Arvense) and Antiretroviral Drugs. *J. Int. Assoc. Provid. AIDS Care* **2017**, *16*, 11–13, doi:10.1177/2325957416680295.
3. The results are clear: for almost any question, answers for the ‘highlighted group’ are more consensual. The only question where it does not apply is M6 and Gs (differences in G are due to differences in type of product selected). This gives us a clue that reading of the article could be by itself a factor of error.

### Respondents agreement scores after modifications – Experts


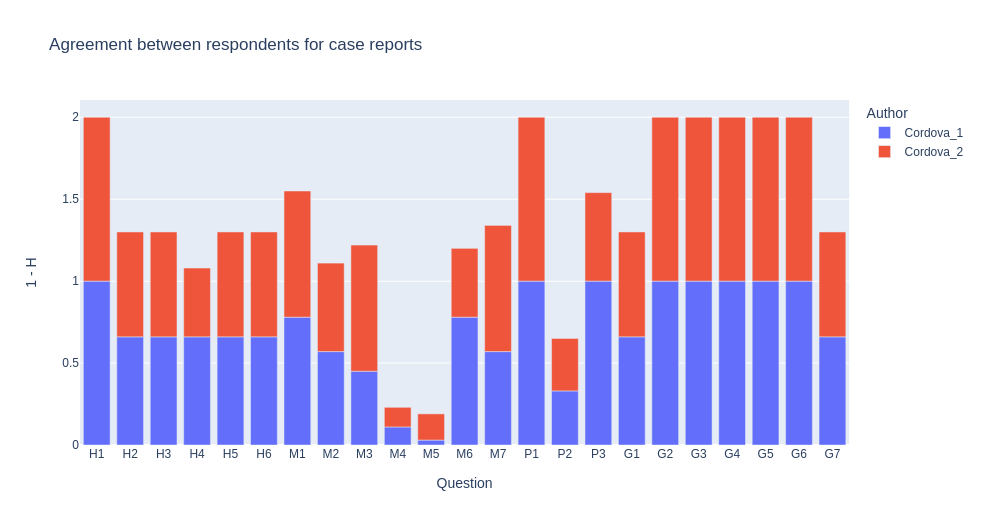
Figure S5: Agreement between respondents for 2 case reports in fourth phase - experts. Agreement was computed as entropy value: $H=-\sum_{i} p_{i}*{log}_{i}\left( p_{i} \right)$, with i the number or possible answers for the question. For clarity purpose, results are presented as 1 - H to assign high values to low entropy. A higher value means a better agreement between respondents; for a single article, a value of 1 means that every respondents gave the same answer while a value of 0 means that they all gave different answers. The bars in red represents answers given by group for whom important information in the article were highlighted while bars in blue represents answers for non-highlighted text. N=17.

### Discussions for the fourth phase of evaluation – Experts:

1. H2-H6: Here, two experts answered that the name of the herbal supplement was mentioned, which is not the case (the article only says, “daily supplements containing horsetail’’ (*Equisetum arvense* L.). The rest of the questions for the herb section was thus also wrong as selecting herbal supplement deactivates the rest of the section.
2. G1 and G7 are again due to different selection of type of product.
3. One person answered ‘no’ for M1 for the two cases. This answer was due to a difference of interpretation of the sentence ‘The study describes an interaction’. Here, the study shows an interaction, but the expert considered the interaction was ‘shown’ but not ‘described’, in the sense that it was not detailed and explained. This is the first time this case occurred for this question, but we decided to change the term ‘describes’ to avoid such cases. Note that it leads to divergences for the rest of the section, as answering ‘no’ deactivates other questions. This expert will thus not be mentioned in further analysis.
4. One person answered ‘yes’ for M2 while all others answered ‘no’. According to this expert, it was a distraction error.
5. In M3, two people answered ‘no’. This case is problematic as these experts answered that the interaction was nor pharmacokinetic nor pharmacodynamic. As we see no case in which it would be possible to answer ‘no’ for both questions, we decided to add a popup warning the respondents on the form.
6. M4-M5: Questions related to metabolic pathways have always been more problematic than others, but it was even worst this time. Here, it is due to a divergence in the sources mentioned: DDI predictor and the article mentioned different pathways for the same combination, leading to completely discordant answers. At first, we decided to use DDI predictor as it provides information above clinically-proved interactions. Yet, due to confusion that can result from cases where the article and DDI predictor diverge, we decided to make the answer dependent only from the article by removing the link to DDI predictor. This has also the advantage to reduce the dependence of the form to external sources.
7. M6-M7 The divergence is due to a single experts answering ‘no’ while others answered ‘yes’.
8. P2: Again, the clinical status of the patient was a source of divergence. This is due to pathology of the patient, which is HIV-positive. This pathology was considered as ‘immunosuppressed’ by some experts, while others considered it as not being listed in the question. As for the grades, this divergence is hard to solve as it would require to be exhaustive in listed pathologies. We decided to leave this as is, as HIV patients with well-balanced treatment should not suffer any specific clinical status as would a patient with listed pathologies justifying a different score.

## Clinical studies form – phase 1

1. During this first round, this first form (Table S5) was designed to assess the important criteria for scoring the imputability of clinical studies.
2. Table S5: First version of the form for clinical studies’ scoring

|  | |  | | |
| --- | --- | --- | --- | --- |
| 1. **N°** | 1. **Item** | 1. **Yes** | 1. **No** | 1. **NA / Unknown** |
| 1. Herb | | | | |
| 1. H1 | 1. The case concerns 3 herbs or more or the patient takes more than 3 drugs | 1. The study cannot be interpreted |  |  |
| 1. H2 | 1. The name of the supplement is mentioned | 1. +2 | 1. 0 | 1. / |
| 1. H3 | 1. The study concerns a single molecule | 1. +1 | 1. 0 | 1. / |
| 1. H4 | 1. If the name of the complement is not mentioned: the latin name of the herb is mentioned | 1. +1 | 1. -1 | 1. / |
| 1. H5 | 1. If the name of the complement is not mentioned: the part of the herb used is mentioned | 1. +1 | 1. -1 | 1. / |
| 1. H6 | 1. If the name of the complement is not mentioned: the extraction solution is mentioned | 1. +1 | 1. -1 | 1. / |
| 1. Mechanism | | | | |
| 1. M1 | 1. The mechanism of interaction is pharmacodynamic | 1. +2 | 1. 0 | 1. 0 |
| 1. M2 | 1. The mechanism of interaction involves a single CYP (or another enzyme or transporter – major pathway) for the herb and the drug | 1. +4 | 1. 0 | 1. 0 |
| 1. M3 | 1. The mechanism of interaction involves a single CYP (or other enzyme or transporter – major pathway) for the herb and the drug is metabolized by multiple isoenzymes – major pathways | 1. -2 | 1. 0 | 1. 0 |
| 1. M4 | 1. The mechanism of interaction involves a drug that is substrate of an isoenzyme for which a polymorphism is probable (CYP2C9, 2C19, 2D6) and the genotype is stated. | 1. -1 | 1. -2 | 1. 0 |
| 1. M5 | 1. The study concerns a single intake of the herb | 1. +3 | 1. 0 | 1. 0 |
| 1. M6 | 1. If and only if an interaction was observed, a rechallenge/dechallenge has been observed | 1. +2 | 1. -4 | 1. -1 |
| 1. M7 | 1. The statistical analysis is coherent with the observation (p≤0.05) | 1. 0 | 1. -2 | 1. 0 |
| 1. Study design | | | | |
| 1. S1 | 1. The cohort of the study includes: | 1. Less than 10 patients +1 | 1. Between 10 and 20 patients +2 | 1. More than 20 patients +3 |
| 1. S2 | 1. The study concerns a single ethnic group | 1. -2 | 1. 0 | 1. -1 |
| 1. S3 | 1. The composition of cohort is biased in terms of sex ratio or age groups | 1. -1 | 1. +4 | 1. 0 |
| 1. S4 | 1. The study concerns healthy patients | 1. +2 | 1. 0 | 1. 0 |
| 1. S5 | 1. The patients suffer a heart/kidney/liver failure, cancer, neurological disorders, immunosuppression, or are transplanted, and the treatment involved in the event is directly related to this pathology | 1. +2 | 1. -2 | 1. 0 |
| 1. S6 | 1. The study lasts more than two weeks | 1. +2 | 1. +1 | 1. 0 |
| 1. S7 | 1. The manufacturer or seller of the herb is an author or co-author | 1. -1 | 1. 0 | 1. 0 |

### Respondents agreement scores

1. For this round table, the experts worked independently on 2 publications:
2. Kim, H.-S.; Kim, G.-Y.; Yeo, C.-W.; Oh, M.; Ghim, J.-L.; Shon, J.-H.; Kim, E.-Y.; Kim, D.-H.; Shin, J.-G. The Effect of Ginkgo Biloba Extracts on the Pharmacokinetics and Pharmacodynamics of Cilostazol and Its Active Metabolites in Healthy Korean Subjects. *Br. J. Clin. Pharmacol.* **2014**, *77*, 821–830, doi:10.1111/bcp.12236.
3. Loughren, M.J.; Kharasch, E.D.; Kelton-Rehkopf, M.C.; Syrjala, K.L.; Shen, D.D. Influence of St. John’s Wort on Intravenous Fentanyl Pharmacokinetics, Pharmacodynamics, and Clinical Effects: A Randomized Clinical Trial. *Anesthesiology* **2020**, *132*, 491–503, doi:10.1097/ALN.0000000000003065.
4. The convergences and discordances can be interpreted in the following figure:

### Discussions for the first phase of evaluation:


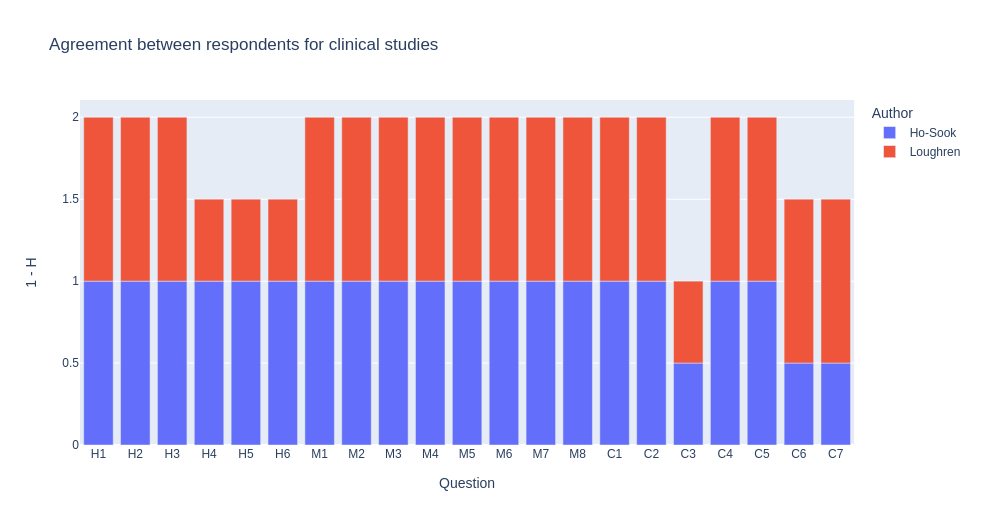
Figure S6: Agreement between respondents for two clinical studies in first phase. Agreement was computed as entropy value: For clarity purpose, results are presented as 1 - H to assign high values to low entropy. A higher value means a better agreement between respondents; for a single article, a value of 1 means that every respondent gave the same answer while a value of 0 means that they all gave different answers. N=4.

1. H4, H5, H6: In Loughren study, some respondents missed information about the herb used (St. John’s wort (one 300-mg Kira tablet; Lichtwer Pharma, Berlin, Germany)). Due to this, the following questions about herb description (that should be skipped due to a complement being cited) suffer some disagreement.
2. S3: The question of the bias is difficult as it is linked to some interpretation. Defining from where start the imbalance would require some arbitrary choice (ex: Loughren - « Sixteen healthy subjects, eight males and eight females, 21 to 41 yr... » is it representative in term of ages?). Here the experts had different interpretations.
3. C6: This question might be ambiguous depending on study protocol. At the beginning, in our mind, it was there to ensure the interaction has time to develop, as for example enzymatic inductions require some days/weeks to take place. In Ho-Sook publication, the study is a two-way crossover study with 2 weeks washout. Technically, it should give a ‘yes’ for the item, but it actually misses the point.
4. C7: Ho-Sook - The study was funded by K Chemicals Co., as it is clearly stated in competing interests section. The question should be clarified to extend beyond authors and co-authors.

## Clinical studies form – phase 2

1. During this second round, this first form (Table S6) was designed to assess the important criteria for scoring the imputability of clinical studies.
2. Table S6: Second version of the form for clinical studies’ scoring

|  | |  | | |
| --- | --- | --- | --- | --- |
| 1. **N°** | 1. **Item** | 1. **Yes** | 1. **No** | 1. **NA / Unknown** |
| 1. Herb | | | | |
| 1. H1 | 1. The study concerns 3 herbs or more ~~or the patient takes more than 3 drugs~~ | 1. / | 1. 0 | 1. 0 |
| 1. H2 | 1. The name of the complement/drug is mentioned | 1. +2 | 1. 0 | 1. 0 |
| 1. H3 | 1. The study concerns a single molecule | 1. +1 | 1. 0 | 1. 0 |
| 1. H4 | 1. ~~If the name of the complement is not mentioned:~~ the latin name of the herb is mentioned | 1. +1 | 1. -1 | 1. 0 |
| 1. H5 | 1. ~~If the name of the complement is not mentioned:~~ the herb part used is mentioned | 1. +1 | 1. -1 | 1. 0 |
| 1. H6 | 1. ~~If the name of the complement is not mentioned:~~ the extraction solution is mentioned | 1. +1 | 1. -1 | 1. 0 |
| 1. Mechanism | | | | |
| 1. M1 | 1. The mechanism of interaction described is pharmacodynamic | 1. +2 | 1. 0 | 1. 0 |
| 1. M2 | 1. The study describes the implication of a single enzyme or transporter (herb) and the drug is substrate of this enzyme or transporter only – major pathway (refer to HUG substrates table or DDI predictor) | 1. +4 | 1. 0 | 1. 0 |
| 1. M3 | 1. The study describes the implication of a single enzyme or transporter (herb) and the drug is substrate of multiple enzyme or transporter – major pathway (refer to HUG substrates table or DDI predictor) | 1. -2 | 1. 0 | 1. 0 |
| 1. M4 | 1. If the drug is a CYP2C9, 2C19 or 2D6 substrate, the patient’s genotype is described | 1. -1 | 1. 0 | 1. 0 |
| 1. M5 | 1. The study describes an event that appeared after a single intake of the herb | 1. +3 | 1. 0 | 1. 0 |
| 1. M6 | 1. If and only if an interaction occurred: a dechallenge or rechallenge was stated | 1. +2 | 1. -4 | 1. -1 |
| 1. M7 | 1. The statistical analysis is coherent with the conclusions (exemple: p-value <= 0.05 if interaction) | 1. 0 | 1. -2 | 1. 0 |
| 1. Cohort | | | | |
| 1. C1 | 1. The cohort of the study includes less than 10 patients | 1. Less than 10 patients +1 | 1. Between 10 and 20 patients +2 | 1. More than 20 patients +3 |
| 1. C2 | 1. The study concerns a single ethnic group | 1. -2 | 1. 0 | 1. -1 |
| 1. C3 | 1. The composition of cohort is biased in terms of sex ratio or age groups | 1. -1 | 1. +4 | 1. 0 |
| 1. C4 | 1. The study concerns healthy patients | 1. +2 | 1. 0 | 1. 0 |
| 1. C5 | 1. If the patients suffer a heart/kidney/liver failure, cancer, neurological disorders, immunosuppression, or are transplanted, the treatment involved in the event is directly related to this pathology | 1. +2 | 1. -2 | 1. 0 |
| 1. C6 | 1. The study lasts more than two weeks | 1. +2 | 1. +1 | 1. 0 |
| 1. C7 | 1. The manufacturer or seller of the herb is cited in the study | 1. -1 | 1. 0 | 1. 0 |

1. For this round table, the experts worked independently on 2 publications:
2. xx
3. yy

## Modifications:

1. H1: Mentions of drugs in this question were removed as the section focuses on description of the herb. As usage of drugs are usually better monitored than herbs, we considered here than the presence of multiple drugs causes less ambiguity than mix of herbs.
2. H4, H5, H5: To clarify questions, the first part of the sentence was removed and the questions where deactivated in the form when ‘yes’ was answered for H2 or H3 to prevent answers. This change responds to case in which users answer to H4, H5 and H6 even though they answered ‘yes’ for H2 or H3.
3. M7: example was clarified by specifying that p-value is valid if an interaction occurred only.
4. C7: The words ‘cited in the study’ have been added instead of ‘author or co-author’ to extend to all situations where a manufacturer could appear if cited.
5. A third round of discussion occurred before testing this iteration of the form.

## Clinical studies form – phase 3

During this second round, this third form (Table S7) was designed to assess the important criteria for scoring the imputability of clinical studies.

Table S7: Third version of the form for clinical studies’ scoring

|  | |  | | |
| --- | --- | --- | --- | --- |
| **N°** | **Item** | **Yes** | **No** | **NA / Unknown** |
| Herb | | | | |
| H1 | The study concerns 3 herbs or more | / | 0 | 0 |
| H2 | The name of the supplement/phytomedicine is mentioned | +2 | 0 | 0 |
| H3 | The study concerns a single molecule | +1 | 0 | 0 |
| H4 | The binomial Latin name of the herb is specified or leaves no ambiguity | +1 | -1 | 0 |
| H5 | The herb part used is mentioned | +1 | -1 | 0 |
| H6 | The extraction solution is mentioned | +1 | -1 | 0 |
| Mechanism | | | | |
| M1 | The study describes an interaction (if the study describes the absence of interaction, select 'no') | 0 | 4 | / |
| M2 | The interaction mechanism described is pharmacodynamic | +5 | 0 | / |
| M3 | The interaction mechanism described is pharmacokinetic | +3 | 0 | / |
| M4 | The study describes the implication of a single enzyme or transporter (herb) and the drug is substrate of this enzyme or transporter only – major pathway (refer to HUG substrates table or DDI predictor) | +4 | 0 | 0 |
| M5 | The study describes the implication of a single enzyme or transporter (herb) and the drug is substrate of multiple enzyme or transporter – major pathway (refer to HUG substrates table or DDI predictor) | -2 | 0 | 0 |
|  | ~~The drug is a CYP2C9, 2C19 or 2D6 substrate and the patient’s genotype is not described~~ | ~~-2~~ | ~~0~~ | ~~0~~ |
| M6 | If the drug is a CYP2C9, 2C19 or 2D6 substrate, the patient’s genotype is described | -1 | -2 | 0 |
| M7 | The study describes an event that appeared after a single intake of the herb | +3 | +2 | 0 |
|  | ~~The study describes an event that appeared after multiple intake of the herb~~ | ~~+2~~ | ~~0~~ | ~~0~~ |
|  | ~~If and only if an interaction occurred: A dechallenge or rechallenge was stated~~ | ~~+2~~ | ~~-4~~ | ~~-1~~ |
| M8 | The statistical analysis is coherent with the conclusions ~~(exemple: p-value <= 0.05 if interaction)~~ | 0 | -2 | 0 |
| Cohort | | | | |
| C1 | The cohort of the study includes | Less than 10 patients: +2 | Between 10 and 20 patients: +3 | More than 20 patients: +4 |
|  | ~~The cohort of the study includes 10 to 20 patients~~ | ~~+2~~ | ~~0~~ | ~~0~~ |
|  | ~~The cohort of the study includes more than 20 patients~~ | ~~+3~~ | ~~0~~ | ~~0~~ |
| C2 | The study concerns a single ethnic group | -2 | 0 | -1 |
| C3 | The composition of cohort is biased in terms of sex ratio or age groups | -1 | +4 | 0 |
| C4 | The study concerns healthy patients | +2 | 0 | 0 |
| C5 | If the patients suffer a heart/kidney/liver failure, cancer, neurological disorders, immunosuppression, or are transplanted, the treatment involved in the event is directly related to this pathology | +2 | -2 | 0 |
|  | ~~The study concerns patients with risk factors not included above~~ | ~~+3~~ | ~~0~~ | ~~0~~ |
| C6 | The study lasts more than 2 weeks | +2 | +1 | 0 |
| C7 | The manufacturer or seller of the herb is an author or co-author | -1 | 0 | 0 |

### Respondents agreement scores – experts

For this round table, the experts worked independently on 2 publications:

1. Walker, A.F.; Marakis, G.; Simpson, E.; Hope, J.L.; Robinson, P.A.; Hassanein, M.; Simpson, H.C.R. Hypotensive Effects of Hawthorn for Patients with Diabetes Taking Prescription Drugs: A Randomised Controlled Trial. *Br. J. Gen. Pract. J. R. Coll. Gen. Pract.* **2006**, *56*, 437–443.
2. Al-Jenoobi, F.I.; Al-Thukair, A.A.; Alam, M.A.; Abbas, F.A.; Al-Mohizea, A.M.; Alkharfy, K.M.; Al-Suwayeh, S.A. Effect of Curcuma Longa on CYP2D6- and CYP3A4-Mediated Metabolism of Dextromethorphan in Human Liver Microsomes and Healthy Human Subjects. *Eur. J. Drug Metab. Pharmacokinet.* **2015**, *40*, 61–66, doi:10.1007/s13318-014-0180-2.

The convergences and discordances can be interpreted in the following figure:

### Discussions for the third phase of evaluation – Experts:


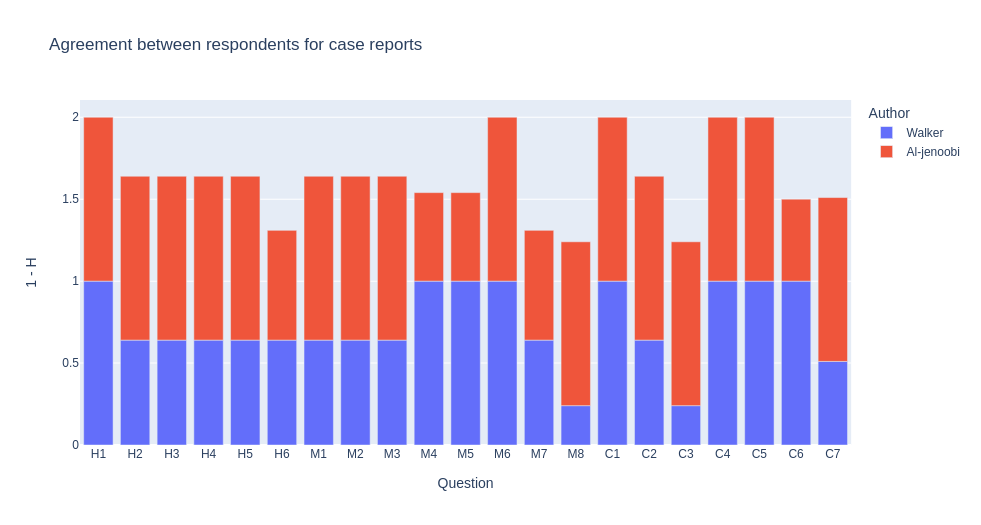
Figure S7: Agreement between respondents for two clinical studies in third phase - experts. Agreement was computed as entropy value: $H=-\sum_{i} p_{i}*{log}_{i}\left( p_{i} \right)$, with i the number or possible answers for the question. For clarity purpose, results are presented as 1 - H to assign high values to low entropy. A higher value means a better agreement between respondents; for a single article, a value of 1 means that every respondents gave the same answer while a value of 0 means that they all gave different answers. N=6.

- H2-6: Again, differences are due to experts missing supplement name while it is mentioned, leading to wrong answers for the rest of the section.
- M1: The question was ambiguous for some respondents; the absence of interaction was interpreted as either ‘the herb doesn’t interact with the drug’ or ‘the article does not talk about a potential interaction between this herb and this drug’. The question was initially thought to be answered as in the former case. Answering ‘no’ leads to skipping the rest of the section until M8, thus leading to divergence to these answers. A reformulation might be needed, but it requires further thinking.
- M8: Finding and understanding the statistical analysis on a clinical study is not an easy task. There is often analysis for multiple parameters along the article, and these are not always easy to interpret experts not used to clinical study design. Yet, the score for this item is not too penalizing, and people interested in assessing the score of a clinical study should be able to answer. As we think this item is too important in assessing imputability, we decided to keep it.
- C6: The was ambiguous again because of study design and washout periods. For now, no solution has been found to clarify the situation.
- C7: The divergence is due to a confusing information in Walker: the competing interests sections states that there are none, while the cited funding body is Lichtwer Pharma UK who funded the study and gave supplements. Some experts answered based only on competing interests while others answer based on this information.

### Respondents agreement scores – students

For this round table, the master students worked independently on the publication:

Kim HS, Kim GY, Yeo CW, et al. The effect of Ginkgo biloba extracts on the pharmacokinetics and pharmacodynamics of cilostazol and its active metabolites in healthy Korean subjects. *Br J Clin Pharmacol*. 2014;77(5):821-830. doi:10.1111/bcp.12236

### Discussions for the third phase of evaluation – Students:


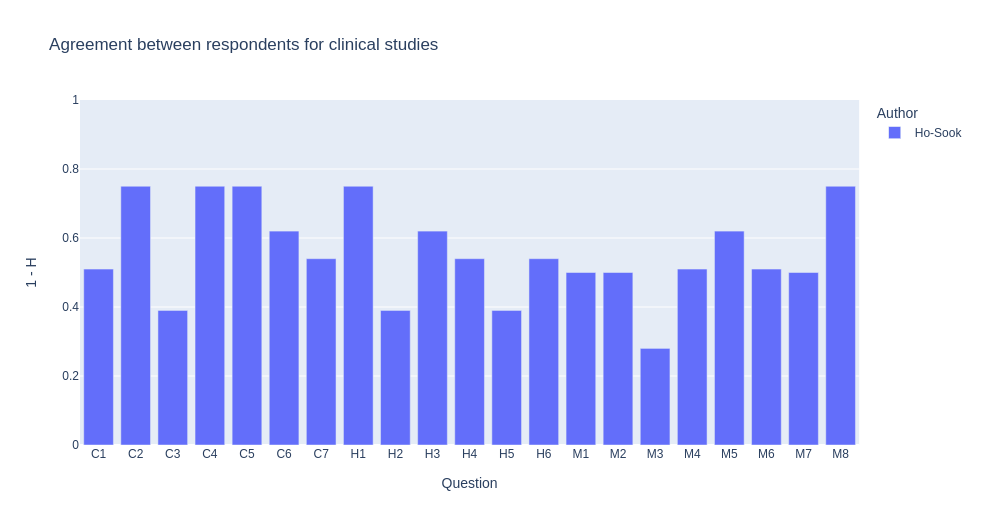
Figure S8: Agreement between respondents for a clinical study in third phase - students. Agreement was computed as entropy value: $H=-\sum_{i} p_{i}*{log}_{i}\left( p_{i} \right)$, with i the number or possible answers for the question. For clarity purpose, results are presented as 1 - H to assign high values to low entropy. A higher value means a better agreement between respondents; for a single article, a value of 1 means that every respondents gave the same answer while a value of 0 means that they all gave different answers. N=9.

The questions that cause most problem are the same as above:

- Herb section caused by the two questions H3 and H4 that deactivates following ones.
- C3, M3

On the overall, student’s answers are worse than experts ones, as excepted due to their inexperience in the field.

## Clinical studies form – phase 4

1. During this second round, this third form (Table S8) was designed to assess the important criteria for scoring the imputability of clinical studies.
2. Table S8: Third version of the form for clinical studies’ scoring

|  | |  | | |
| --- | --- | --- | --- | --- |
| 1. **N°** | 1. **Item** | 1. **Yes** | 1. **No** | 1. **NA / Unknown** |
| 1. Herb | | | | |
| 1. H1 | 1. The study concerns 3 herbs or more | 1. / | 1. 0 | 1. 0 |
| 1. H2 | 1. The name of the complement/phytomedicine is mentioned | 1. +2 | 1. 0 | 1. 0 |
| 1. H3 | 1. The study concerns a single molecule | 1. +1 | 1. 0 | 1. 0 |
| 1. H4 | 1. The binomial Latin name of the herb is specified or leaves no ambiguity | 1. +1 | 1. -1 | 1. 0 |
| 1. H5 | 1. The herb part used is mentioned | 1. +1 | 1. -1 | 1. 0 |
| 1. H6 | 1. The extraction solution is mentioned | 1. +1 | 1. -1 | 1. 0 |
| 1. Mechanism | | | | |
| 1. M1 | 1. The study describes an interaction (if the study describes the absence of interaction, select 'no') | 1. 0 | 1. 4 | 1. / |
| 1. M2 | 1. The interaction mechanism described is pharmacodynamic | 1. +5 | 1. 0 | 1. / |
| 1. M3 | 1. The interaction mechanism described is pharmacokinetic | 1. +3 | 1. 0 | 1. / |
| 1. M4 | 1. The study describes the implication of a single enzyme or transporter (herb) and the drug is substrate of this enzyme or transporter only – major pathway (refer to HUG substrates table or DDI predictor) | 1. +4 | 1. 0 | 1. 0 |
| 1. M5 | 1. The study describes the implication of a single enzyme or transporter (herb) and the drug is substrate of multiple enzyme or transporter – major pathway (refer to HUG substrates table or DDI predictor) | 1. -2 | 1. 0 | 1. 0 |
| 1. M6 | 1. If the drug is a CYP2C9, 2C19 or 2D6 substrate, the patient’s genotype is described | 1. -1 | 1. -2 | 1. 0 |
| 1. M7 | 1. The study describes an event that appeared after a single intake of the herb | 1. +3 | 1. +2 | 1. 0 |
| 1. M8 | 1. The statistical analysis is coherent with the conclusions | 1. 0 | 1. -2 | 1. 0 |
| 1. Cohort | | | | |
| 1. C1 | 1. The cohort of the study includes less than 10 patients | 1. +1 | 1. +2 | 1. / |
| 1. C2 | 1. The cohort of the study includes more than 20 patients | 1. +2 | 1. 0 | 1. / |
| 1. C3 | 1. The study concerns a single ethnic group | 1. -2 | 1. 0 | 1. -1 |
| 1. C4 | 1. The composition of cohort is biased in terms of sex ratio or age groups | 1. -1 | 1. +2 | 1. 0 |
| 1. C5 | 1. The study concerns healthy patients | 1. +2 | 1. 0 | 1. 0 |
| 1. C6 | 1. If the patients suffer a heart/kidney/liver failure, cancer, neurological disorders, immunosuppression, or are transplanted, the treatment involved in the event is directly related to this pathology | 1. +2 | 1. -2 | 1. 0 |
| 1. C7 | 1. The study lasts more than 2 weeks | 1. +2 | 1. +1 | 1. 0 |
| 1. C8 | 1. The manufacturer or seller of the herb is an author or co-author | 1. -1 | 1. 0 | 1. 0 |

### Respondents agreement scores after modifications – Students

1. For this round table, the 13 master students worked independently on the publication:
2. Kim HS, Kim GY, Yeo CW, et al. The effect of Ginkgo biloba extracts on the pharmacokinetics and pharmacodynamics of cilostazol and its active metabolites in healthy Korean subjects. *Br J Clin Pharmacol*. 2014;77(5):821-830. doi:10.1111/bcp.12236

### Discussions for the fourth phase of evaluation – Students:


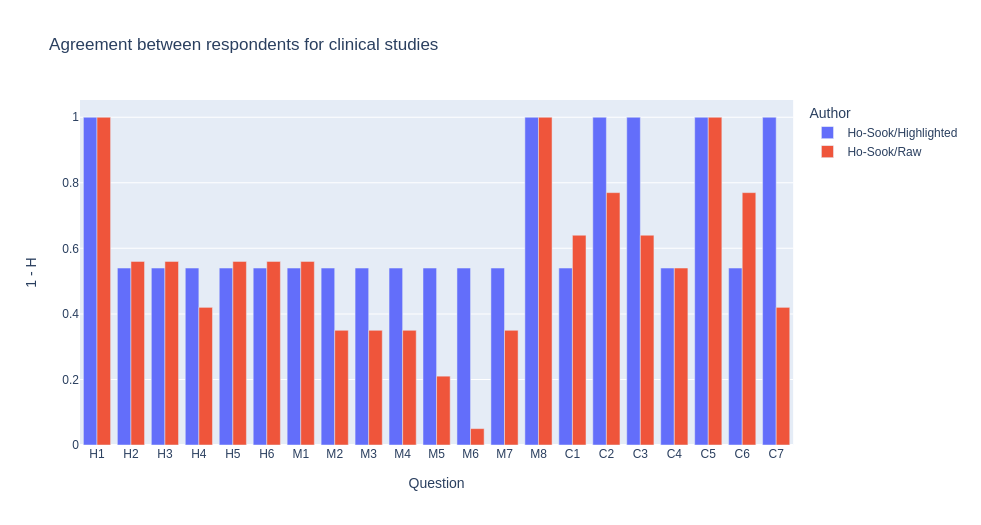
Figure S9: Agreement between respondents for a clinical study in fourth phase - students. Agreement was computed as entropy value: $H=-\sum_{i} p_{i}*{log}_{i}\left( p_{i} \right)$, with i the number or possible answers for the question. For clarity purpose, results are presented as 1 - H to assign high values to low entropy. A higher value means a better agreement between respondents; for a single article, a value of 1 means that every respondents gave the same answer while a value of 0 means that they all gave different answers. The bars in red represents answers given by group for whom important information in the article were highlighted while bars in blue represents answers for non-highlighted text. N=13.

1. During this phase, we decided to test the impact of focus on the score. We already noted that filling the form takes time and require the user to find some specific pieces of information. Besides understanding of the question, one important factor that can lead to wrong answers is the lack of focus, people reading the article diagonally, or missing some “hidden” information (numbers in tables, information in footnotes, …). To try to objectify this effect, we divided a pool of students in two groups: one group received highlighted article while the other group received the same article without any annotation.
2. The results are clear: for almost any question, answers for the ‘highlighted group’ are more consensual. The only question where it does not apply is M6 and Gs (differences in G are due to differences in type of product selected). This gives us a clue that reading of the article could be by itself a factor of error.

### Respondents agreement scores after modifications – Experts

1. For this round table, experts worked independently on the publication:
2. Bell, E.C.; Ravis, W.R.; Lloyd, K.B.; Stokes, T.J. Effects of St. John’s Wort Supplementation on Ibuprofen Pharmacokinetics. *Ann. Pharmacother.* **2007**, *41*, 229–234, doi:10.1345/aph.1H602.

### Discussions for the fourth phase of evaluation – Experts:


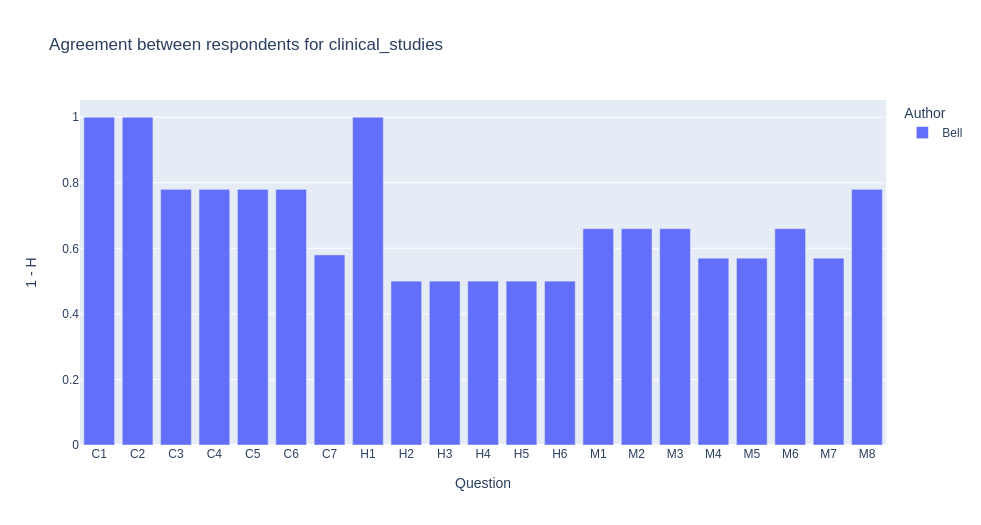


Figure S10: Agreement between respondents for a clinical study in fourth phase - experts. Agreement was computed as entropy value: $H=-\sum_{i} p_{i}*{log}_{i}\left( p_{i} \right)$, with i the number or possible answers for the question. For clarity purpose, results are presented as 1 - H to assign high values to low entropy. A higher value means a better agreement between respondents; for a single article, a value of 1 means that every respondents gave the same answer while a value of 0 means that they all gave different answers. The bars in red represents answers given by group for whom important information in the article were highlighted while bars in blue represents answers for non-highlighted text. N=13.

1. While there is still some disagreement for the same questions as before, scores tend to be more robust than in previous iterations. In agreement with the experts, this latest version has been validated and will remain unchanged.

# Grade Evaluation

1. To assess the risk of an IHD, we focused on 2 types of scores. The first concerns the clinical/physiological observation used to detect it. We call this first stage the clinical risk. To assess it, we first asked the scoreers to classify this risk by following the information in a table (see below).
2. Table S9: Grades and their associated manifestation

| 1. Grade | 1. Manifestation |
| --- | --- |
| 1. Grade 0 | 1. No or nonsignificant or negligible clinical symptom and AUC variation under 30%, INR under 4, ... |
| 1. Grade 1 | 1. No or nonsignificant or negligible clinical symptom but AUC variation above 30%, INR above 4, ... |
| 1. Grade 2 | 1. Mild symptoms for <2 days: fatigue, nausea, headache, ... |
| 1. Grade 3 | 1. Moderate symptoms for 2-7 days;  Loss of effectiveness for the treatment of a non-serious disease or residual symptoms or invalidity |
| 1. Grade 4 | 1. Severe symptoms for >7 days;  Loss of effectiveness for the treatment of a serious disease not deadly in short term |
| 1. Grade 5 | 1. Increase of death risk,  Loss of effectiveness for the treatment of a serious disease deadly in short term,  Increase of pregnancy risk without risk factor for mother or child |
| 1. Grade 6 | 1. Death; increase of pregnancy risk with risk factor for mother or child |

## Limitation of the grades table:

1. Handling changes in parameters is tricky, as it leads to a lot of interpretation. At first, Grade 1 tends to shunt the higher grades due to being too general. For example, an AUC increased by 100% for an anticoagulant drug would technically fit in grade 1 but could actually cause severe troubles. Besides, it is impossible to give a threshold value for all existing parameters, and these thresholds will always be arbitrary.
2. Use of symptoms duration as a criterion is also problematic. While it might make sense at first sight and is associated with severity gradation (severe symptoms for >7 days, …), counterexample exist. For example, a stroke occurs in some hours, while a cold can last for 2 weeks.
3. The terms ‘short-term’ and ‘serious/non-serious’ are also subject to interpretation. Again, tackling the problem would require listing every possible pathology/symptom, which is not realist and would still be arbitrary. As a comparison, the CTCAE provides a well-build grading system, but is composed of more than 150 pages of tables. Using it would add an extreme mental cost to the form.
4. As a possible solution to optimize the score, a rework of the table on the form of a flowchart has been designed.

### Flowchart

### A first version of the flowchart has been designed. It's simpler and forces the scorer to go through all the steps to give his score.


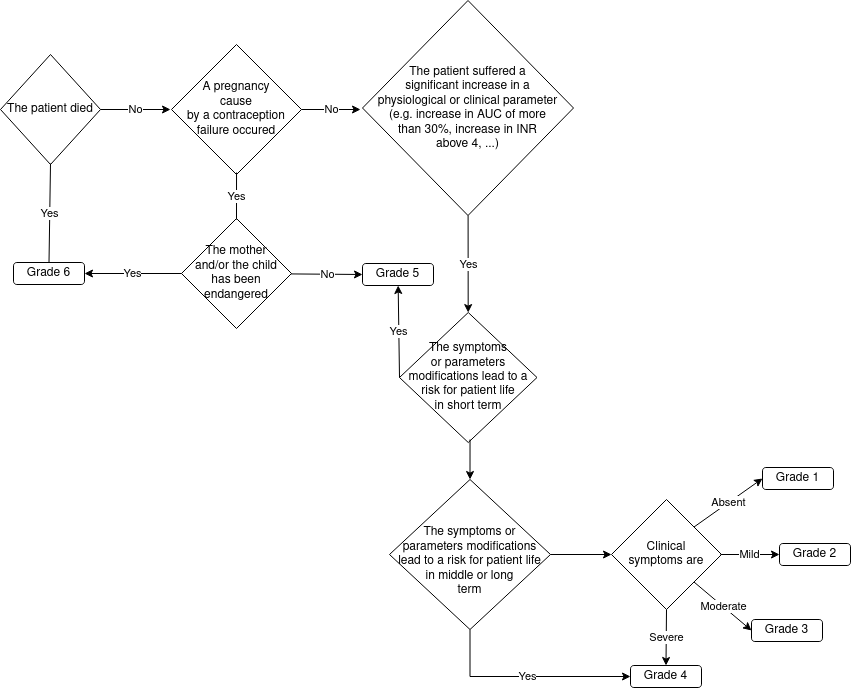
Figure S11: Flowchart for grade selection.

### Comparison between flowchart and table

1. At this stage, we divided the scoring group into 2. They worked on the same 10 pieces of data. Group A scored texts from 1 to 5 using the table and texts from 6 to 10 using the flowchart. Group B scored texts from 1 to 5 using the flowchart and texts from 6 to 10 using the table. The results are shown below:
2. As shown in Figure S12, no real difference in agreement is observed between table and flowchart, with some texts being more consensual with flowchart while others are more consensual with table. According to experts, flowchart is easier to use and helps to avoid missing criteria compared to the table.


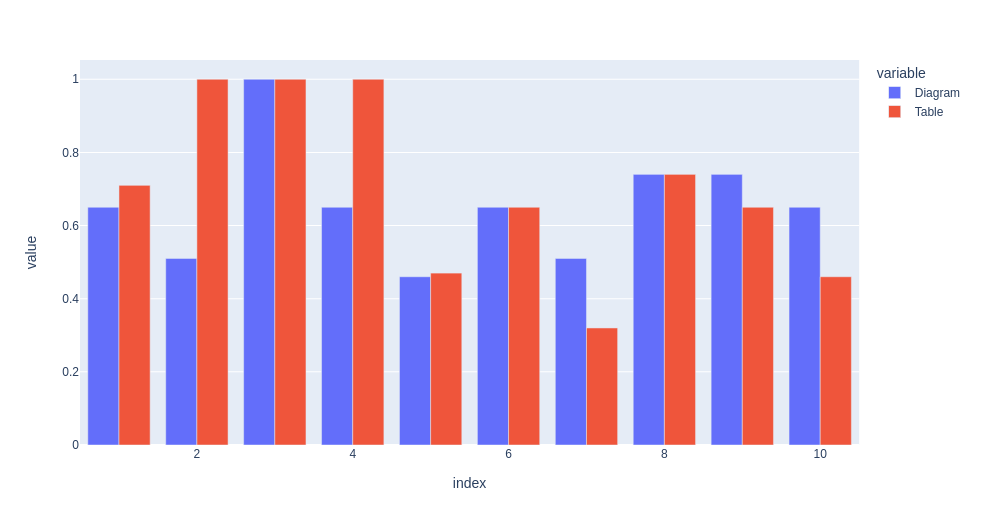
Figure S12: Agreement between respondents for severity of a set of interactions descriptions. Agreement was computed as entropy value: $H=-\sum_{i} p_{i}*{log}_{i}\left( p_{i} \right)$, with i the number or possible answers for the question. For clarity purpose, results are presented as 1 - H to assign high values to low entropy. A higher value means a better agreement between respondents; for a single article, a value of 1 means that every respondents gave the same answer while a value of 0 means that they all gave different answers. The bars in red represents answers given by group for whom important information in the article were highlighted while bars in blue represents answers for non-highlighted text. N=10.
